# rWTC-MBTA: autologous vaccine prevents metastases via antitumor immune responses

**DOI:** 10.1186/s13046-023-02744-8

**Published:** 2023-07-12

**Authors:** Juan Ye, Herui Wang, Rogelio Medina, Samik Chakraborty, Mitchell Sun, Alex Valenzuela, Xueyu Sang, Yaping Zhang, Ondrej Uher, Jan Zenka, Karel Pacak, Zhengping Zhuang

**Affiliations:** 1 Neuro-Oncology Branch, Center for Cancer Research, National Cancer Institute, National Institutes of Health, Building 37, Room 1000 37 Convent Dr, Bethesda, MD 20892 USA; 2 NE1 Inc, New York, NY USA; 3 David Geffen School of Medicine, University of California, Los Angeles, CA USA; 4 Charles R. Drew University of Medicine and Science, Los Angeles, CA USA; 5 Section on Medical Neuroendocrinology, Eunice Kennedy Shriver National Institute of Child Health and Human Development, National Institutes of Health, Bethesda, MD USA; 6 Department of Medical Biology, Faculty of Science, University of South Bohemia, České Budějovice, Czech Republic

**Keywords:** Mannan-BAM, TLR agonists, T-cell cytotoxicity, Metastasis, rWTC-MBTA vaccine

## Abstract

**Background:**

Autologous tumor cell-based vaccines (ATVs) aim to prevent and treat tumor metastasis by activating patient-specific tumor antigens to induce immune memory. However, their clinical efficacy is limited. Mannan-BAM (MB), a pathogen-associated molecular pattern (PAMP), can coordinate an innate immune response that recognizes and eliminates mannan-BAM-labeled tumor cells. TLR agonists and anti-CD40 antibodies (TA) can enhance the immune response by activating antigen-presenting cells (APCs) to present tumor antigens to the adaptive immune system. In this study, we investigated the efficacy and mechanism of action of rWTC-MBTA, an autologous whole tumor cell vaccine consisting of irradiated tumor cells (rWTC) pulsed with mannan-BAM, TLR agonists, and anti-CD40 antibody (MBTA), in preventing tumor metastasis in multiple animal models.

**Methods:**

The efficacy of the rWTC-MBTA vaccine was evaluated in mice using breast (4T1) and melanoma (B16-F10) tumor models via subcutaneous and intravenous injection of tumor cells to induce metastasis. The vaccine’s effect was also assessed in a postoperative breast tumor model (4T1) and tested in autologous and allogeneic syngeneic breast tumor models (4T1 and EMT6). Mechanistic investigations included immunohistochemistry, immunophenotyping analysis, ELISA, tumor-specific cytotoxicity testing, and T-cell depletion experiments. Biochemistry testing and histopathology of major tissues in vaccinated mice were also evaluated for potential systemic toxicity of the vaccine.

**Results:**

The rWTC-MBTA vaccine effectively prevented metastasis and inhibited tumor growth in breast tumor and melanoma metastatic animal models. It also prevented tumor metastasis and prolonged survival in the postoperative breast tumor animal model. Cross-vaccination experiments revealed that the rWTC-MBTA vaccine prevented autologous tumor growth, but not allogeneic tumor growth. Mechanistic data demonstrated that the vaccine increased the percentage of antigen-presenting cells, induced effector and central memory cells, and enhanced CD4^+^ and CD8^+^ T-cell responses. T-cells obtained from mice that were vaccinated displayed tumor-specific cytotoxicity, as shown by enhanced tumor cell killing in co-culture experiments, accompanied by increased levels of Granzyme B, TNF-α, IFN-γ, and CD107a in T-cells. T-cell depletion experiments showed that the vaccine’s antitumor efficacy depended on T-cells, especially CD4^+^ T-cells. Biochemistry testing and histopathology of major tissues in vaccinated mice revealed negligible systemic toxicity of the vaccine.

**Conclusion:**

The rWTC-MBTA vaccine demonstrated efficacy in multiple animal models through T-cell mediated cytotoxicity and has potential as a therapeutic option for preventing and treating tumor metastasis with minimal systemic toxicity.

**Supplementary Information:**

The online version contains supplementary material available at 10.1186/s13046-023-02744-8.

## Background

Tumor metastasis poses a significant challenge for clinical cancer management. Metastatic cancer cells frequently resist conventional therapies and have limited treatment options available [1]. Despite surgical procedures, radiation therapy, chemotherapy, and targeted therapies, the incidence of metastasis remains high [2]. Standard postoperative treatments can only be administered for a limited period after tumor resection and often fail to eliminate metastatic cells or ensure long-term treatment efficacy [3]. As a result, patients with metastatic tumors have a poor prognosis. They frequently experience recurrence shortly after the initial tumor resection, with a lower likelihood of achieving long-term remission than patients with localized cancer [4, 5].

Immunotherapy represents a new therapeutic approach that harnesses the immune system’s capabilities to combat cancer [6]. Cancer vaccines have garnered significant interest as a form of immunotherapy with the potential to provide sustained clinical benefits in inhibiting tumor growth, recurrence, and metastasis [7, 8]. Among cancer vaccines, autologous tumor cell-based vaccines (ATVs) have emerged as a promising therapeutic because they elicit strong antitumor immune responses by harnessing patient-specific tumor antigens to activate T-helper and cytotoxic lymphocytes [9, 10]. This, in turn, induces durable immune memory, thereby offering long-term protection against micrometastatic tumor cells [11]. Despite their promise, the efficacy of ATVs in clinical practice remains limited. This is likely due to several factors, including the low immunogenicity of tumor cells, an immunosuppressive tumor microenvironment, and inconsistent vaccine processing [12]. This highlights the need for innovative strategies to enhance the efficacy of ATVs and target metastatic tumors.

**Fig. 1:**
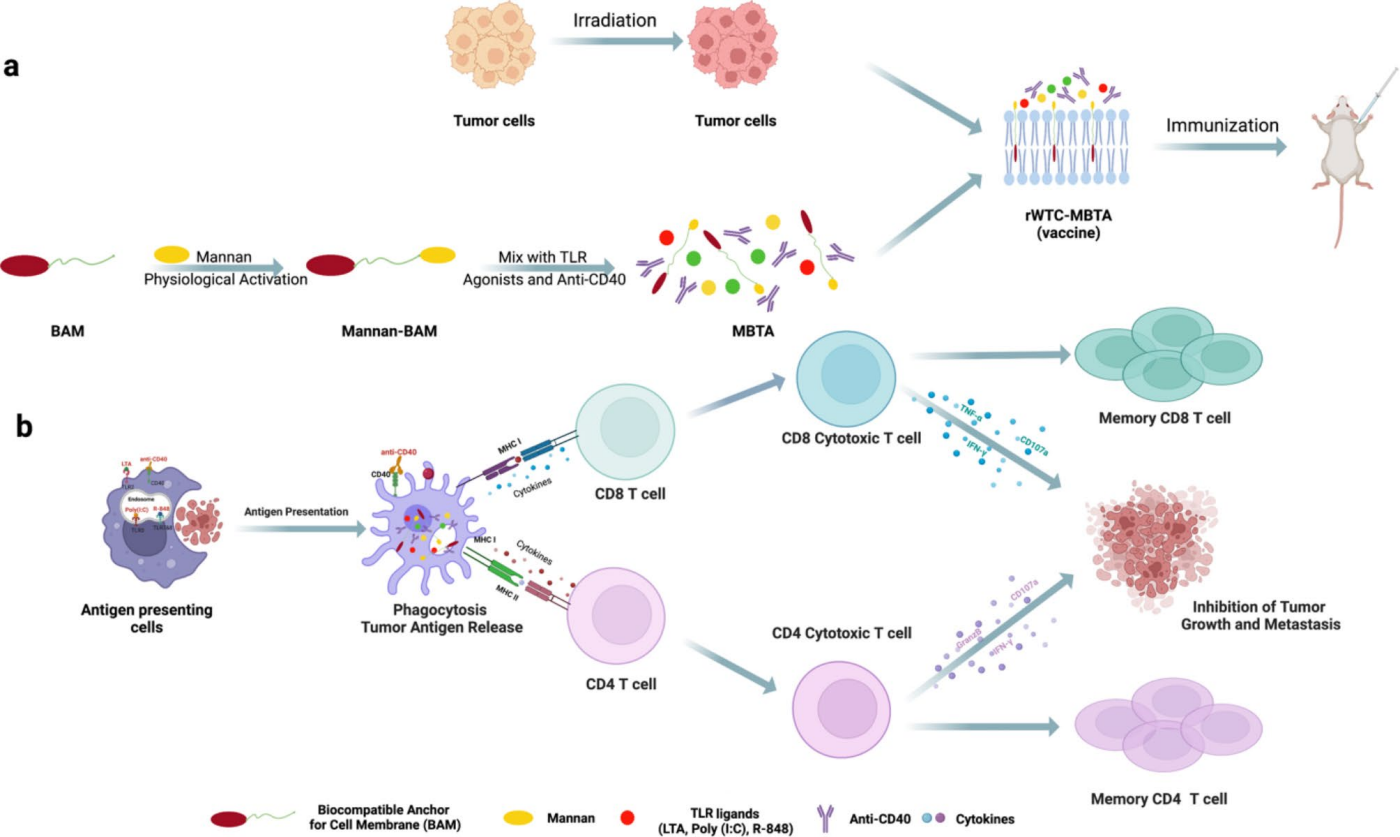
Schematic illustration of rWTC-MBTA vaccine for cancer immunotherapy (by Biorender). **a**, Fabrication process of the rWTC-MBTA vaccine. **b**, Simplified mechanism of MBTA-mediated cancer immunotherapy to prevent post-operative tumor recurrence and metastasis

We have previously demonstrated the therapeutic efficacy of an autologous whole tumor cell vaccine composed of irradiated entire tumor cells (rWTC) pulsed with mannan-BAM, TLR agonists, and anti-CD40 antibody (MBTA) (Fig. 1). This strategy was effective at preventing primary tumor growth and enhancing mouse survival in a colon carcinoma model [13]. Drawing upon these compelling results, we recognized the critical need for preventive strategies in combatting metastatic disease. Thus, in this study we sought to determine the efficacy of rWTC-MBTA as a preventative vaccine in multiple metastasis animal models.

This vaccine is designed to convert immunologically “cold” into “hot” tumors by eliciting a potent innate and adaptive immune response against tumor cells. Mannan-BAM, a pathogen-associated molecular pattern (PAMP), plays a crucial role in coordinating an innate immune response that recognizes and eliminates mannan-BAM-labeled tumor cells [14–16]. Additionally, TLR agonists and anti-CD40 antibodies reinforce the immune response by activating antigen-presenting cells (APCs) to present tumor antigens to the adaptive immune system [17, 18]. This synergistic approach together enhances the anti-tumor immune response.

Herein we demonstrate the efficacy of the rWTC-MBTA vaccine in preventing tumor metastasis and recurrence in various animal models, including tail vein metastatic breast, subcutaneous breast, and tail vein metastatic melanoma cancer models. Notably, we have also shown that administering the vaccine during the critical window of early tumor seeding, after surgical resection of the primary tumor, effectively prevented early metastatic disease in a postoperative metastatic model that closely mimics the clinical setting.

The findings of our study highlight the potential of the rWTC-MBTA vaccine as a vaccine to prevent and treat metastatic tumors. This strategy holds significant implications for cancer immunotherapy and the development of more effective treatments for metastatic disease, addressing the limitations of current approaches and paving the way for improved clinical outcomes.

## Methods

### Materials

The Biocompatibility Anchors for Cell Membranes (BAM) were acquired from NOF America (**White Plains, NY**). Mannan from Saccharomyces cerevisiae, polyinosinic-polycytidylic acid sodium salt, and lipoteichoic acid from Bacillus subtilis were obtained from Sigma-Aldrich (**St. Louis, MO**). Resiquimod (R-848) was purchased from Tocris Bioscience (**Minneapolis, MN**). Anti-mouse CD40 (clone: FGK4.5/GFK45) was procured from BioXCell (**West Lebanon, NH**). Flow cytometry antibodies, including anti-CD4, anti-CD8, anti-CD11c, anti-IFN-γ, anti-CD44, anti-CD62L, anti-Ly6G, anti-Ly6C, anti-CD11b were obtained from BD biosciences (**Franklin Lakes, NJ**); anti-CD45, anti-CD107a, anti-CD80, anti-CD86, anti-CD137L, anti-MHCII, anti-TNF-α, anti-CD19, anti-MHCII, anti-TCRβ and anti-GranB were obtained from Bioligand (**San Diego, CA**). The ELISA kits for detecting IFN-γ, TNF-α, and IL-6 were obtained from Bioligand (**San Diego, CA**).

### Cell lines

The 4T1 mouse mammary tumor cells and EMT6 mouse mammary tumor cells were procured from ATCC and were maintained in RPMI 1640 medium supplemented with 10% v/v fetal bovine serum (FBS). B16-F10 melanoma tumor cells were obtained from ATCC and cultured in DMEM supplemented with 10% v/v fetal bovine serum (FBS) and 1% penicillin/streptomycin. All media, penicillin/streptomycin antibiotics, and FBS were procured from Gibco (**Gaithersburg, MD**).

### Animals

Healthy female BALB/c mice (6–8 weeks) and C57BL/6 mice (6–8 weeks) were procured from Charles River Laboratories (**Wilmington, MA**). All animal experiments were conducted with the approval of the NCI Animal Use and Care Committee. The NCI-CCR affiliated staff performed all animal procedures reported in this study. They were approved by the NCI Animal Care and Use Committee (ACUC) following federal regulatory requirements and standards. AAALAC International accredited all aspects of the intramural NIH ACU program.

### rWTC-MBTA vaccine preparation

There are two steps in vaccine preparation. The first step involves the synthesis of MBTA. We synthesized Mannan-BAM as previously reported [13]. We prepared aminated mannan using a reductive amination method [19]. A solution of Mannan in ammonium acetate (300 mg/ml) was subjected to reduction with 0.2 M sodium cyanoborohydride at pH 7.5 and 50 °C for 5 days. The resulting solution was dialyzed overnight against PBS at 4 °C using MWCO 3500 dialysis tubing (**Serva, Heidelberg, Germany**). According to Kato et al. [20], BAM binds to the amino group of mannoprotein at pH 7.3. The N-hydroxysuccinimide (NHS) group of BAM was allowed to react with the amino group of mannan at room temperature for 1 h. The conjugated solution was then dialyzed against PBS at 4 °C overnight using MWCO 3500 dialysis tubing (**Serva, Heidelberg, Germany**) to form Mannan-BAM (MB). The MBTA solution was prepared using the following formula:50 µL of 0.2 mM Mannan-BAM solution containing 25 µg R-848 (hydrochloric acid form), 25 µg poly(I:C), 25 µg LTA, and 1 µg anti-CD40. The resulting solution was filtered using a 0.22 μm filter and stored at -20 °C until use.

The next step involves irradiating the tumor cells and combining the components. To obtain irradiated whole tumor cells (1 × 10^6^ tumor cells suspended in 50 𝜇L PBS for each mouse dose), they were exposed to 100 Gy using a ^137^Cs MARK I model irradiator (**JL Shepherd & Associates, San Fernando, CA**). After irradiation, the tumor cells (50 𝜇L, per mouse) were incubated with MBTA (50 𝜇L/ mouse) for 0.5-1 h. Subsequently, the rWTC-MBTA vaccine (100 𝜇L per mouse) was injected subcutaneously in the right flank of the treated animals according to the prescribed treatment schedule.

### rWTC-MBTA induced immune response in vivo

To investigate the immunogenicity of the rWTC-MBTA vaccine, we immunized groups of mice with r4T1-MBTA, r4T1, MBTA-only, or PBS. On days 5 and 26, lymph nodes were collected from the immunized mice and dissociated them into single cell suspension, which were stained with anti-CD45, dump channel (anti-CD3/CD19/Ter119/NK1.1), anti-CD11c, CD11b, Ly6G, Ly6C, MHCII, anti-CD80, and anti-CD86 antibodies. CD45^+^MHCII^+^CD11c^+^ antibodies were gated as dendritic cells, while anti-CD80^+^anti-CD86^+^ dendritic cells were gated as mature dendritic cells; CD45^+^CD11c^−^CD11b^+^Ly6G^−^Ly6C^+^ was gated as monocytes; CD45^+^CD11c^−^CD11b^+^Ly6G^−^Ly6C^+^MHCII^+^ was gated as MHCII^+^ monocytes; CD45^+^TCRβ^−^CD19^+^ was gated as B cells; and CD45^+^TCRβ^−^CD19^+^CD137L^+^ was gated as activated B cells. Flow cytometry was performed using a FACSymphony (BD Biosciences).

To evaluate the immune response induced by vaccination, serum samples were collected from immunized mice on days 5 and 26 post-vaccination. The concentrations of IFN-γ, TNF-α, and IL-6 were measured in the collected serum samples using ELISA kits from Bioligand (**San Diego, CA**), following the manufacturer’s protocol.

### Antitumor metastatic effect of MBTA vaccine

This study aimed to investigate the antimetastatic effect of the r4T1-MBTA vaccine by establishing two metastatic animal models: melanoma B16-F10 and breast tumor 4T1 syngeneic metastasis animal models. Mice were randomly divided into four treatment groups: control (immunized with normal PBS), MBTA only, irradiated cells only (r4T1 or rB16-F10), and vaccine (r4T1-MBTA or rB16-MBTA). The immunizations were administered daily for three days, followed by weekly immunizations for three weeks (nine injections). One week after complete immunization, 4T1 cells (1 × 10^5^) or B16-F10 cells (1 × 10^5^) were suspended in 100 µL PBS and injected via the tail vein to establish metastatic animal models. The mice were monitored for the next two weeks to evaluate their health status. At the end of the study, peripheral blood samples, lung/heart/liver/kidney tissue, lymph nodes, and spleen were collected for analysis.

For tumor resection studies, BALB/c mice were implanted subcutaneously with 1 × 10^5^ 4T1 tumor cells into the right No. 3 mammary fat pad. After approximately ten days when the tumor size reached around 50 mm^3^, the mice were randomly divided into two groups (control, and r4T1-MBTA vaccine). The tumors were surgically resected, and the mice received PBS or r4T1-MBTA vaccine according to a 4-week schedule with three injections per week. For the dynamic study, we euthanized three mice per group and collected the lungs on days 0, 7, 14, 21 and 28 post-tumor surgery. The health status of the mice was monitored daily, and their survival was closely monitored. Two weeks after tumor implantation, lung tissues were collected from half of the mice for H&E staining, while the remaining half of the mice were observed for survival curves.

In the immune T cell depletion studies, mice were immunized with PBS or the r4T1-MBTA vaccine and then the immunized mice were divided into three groups: r4T1-MBTA vaccine, CD8 depletion, and CD4 depletion. Three days after the final dose of the vaccine, mice in depleted group were injected with 250 µg of either CD8-depleting antibody (**clone 53 − 6.7; BioXcell**) or CD4-depleting antibody (**clone GK1.5; BioXcell**) on days − 2, -1, and 0 (before tumor implantation), and then weekly thereafter.

### Antitumor studies using an autologous tumor model

To investigate the antitumor immune response, we immunized BALB/c mice with r4T1-MBTA, r4T1, MBTA only, or PBS by performing immunizations for four weeks with three injections per week. One week after immunization with vaccine, mice were inoculated with 2 × 10^5^ 4T1 cells. Two weeks post-inoculation, peripheral blood samples, lymph nodes, and spleens were collected. The spleens were manually ground and filtered through a 70 μm MACS SmartStrainer (**Miltenyi Biotec, Inc**.) for tissue dissociation. Red blood cells were lysed using ACK lysis buffer, and were washed once with PBS, and centrifuged for 10 min at 500×*g*. Isolated cells were divided into two fractions, with one fraction incubated with anti-TCRβ, anti-CD45, anti-CD4, anti-CD8, anti-CD44, and anti-CD62L antibodies. Flow cytometry was used to analyze percentage of immune cells and effector/central memory cells. According to a previous study [21, 22], another fraction of cells was co-cultured with 4T1 tumor cells at a ratio of 10:1. After 48 h of co-culture, the supernatant was collected for the analysis of cytokines IFN-γ and TNF-α using ELISA. The co-cultured cells were incubated with anti-CD4, anti-CD8, anti-GranB, anti-CD107, IFN-γ and TNF-α antibodies for immune functional analysis. Tumor cells were counted using counting beads (**Life Technologies| AB / Invitrogen, Waltham**).

### Antitumor studies using an allogeneic tumor model

To investigate the potential of r4T1-rWTC-MBTA vaccines in personalized immunotherapy, we conducted antitumor studies using an allogeneic tumor model. Initially, BALB/c mice were immunized with r4T1-MBTA, r4T1, MBTA only, or PBS. Immunization was administered for four weeks, with three injections per week. One week after the final vaccination, immunized mice were subcutaneously injected with 1 × 10^5^ EMT6 cells. The health status and tumor growth of the mice were monitored for two weeks. At the endpoint of the study, we collected the lungs from each group for metastasis examination by H&E staining.

### Long-term effects of rWTC-MBTA vaccines induced anti-metastasis in vivo

To investigate the long-term immunological memory effects of the rWTC-MBTA vaccine, we immunized BALB/c mice with r4T1-MBTA, r4T1, MBTA only, or PBS. The immunization schedule involved immunizations for four weeks with three injections per week. Three months (90 days) after the first immunization, we established a lung metastasis model by intravenously injecting the immunized mice with 1 × 10^5^ 4T1 tumor cells. The mice were monitored for two weeks and then euthanized. The establishment of 4T1 lung metastases was examined by H&E staining.

### Toxicity evaluation

To investigate the safety of the r4T1-MBTA vaccine, healthy BALB/c mice were randomly divided into four groups. Mice in each group were immunized with r4T1-MBTA, r4T1, MBTA only, or PBS. Blood samples were collected from each mouse three days after the first dose of immunization and three days after the last vaccination for biochemical analysis. Biochemical parameters, including aspartate aminotransferase (AST), alanine aminotransferase (ALT), cholesterol, triglycerides, amylase, glucose, blood urea nitrogen (BUN), and uric acid were measured to evaluate major organ function. In addition, major organs such as the liver, spleen, heart, kidney, and lung were excised, fixed with 4% paraformaldehyde, and sectioned for H&E staining to assess any tissue damage or abnormalities.

### Statistical analysis

Multiple group comparisons were performed using ordinary one-way ANOVA with Ordinary one-way Multiple comparisons. The unpaired T-test was used to assess the significance of differences between the two groups. The survival rate was analyzed using the Mantel-Cox (log-rank) test. Statistical analyses and figures were conducted using GraphPad Prism 8.4.0 (**GraphPad Software, CA, USA**). All data were represented as mean ± SD. P-values < 0.05 were considered statistically significant.

## Results

### The rWTC-MBTA vaccine prevents tumor metastasis in breast and melanoma metastasis animal models

**Fig. 2:**
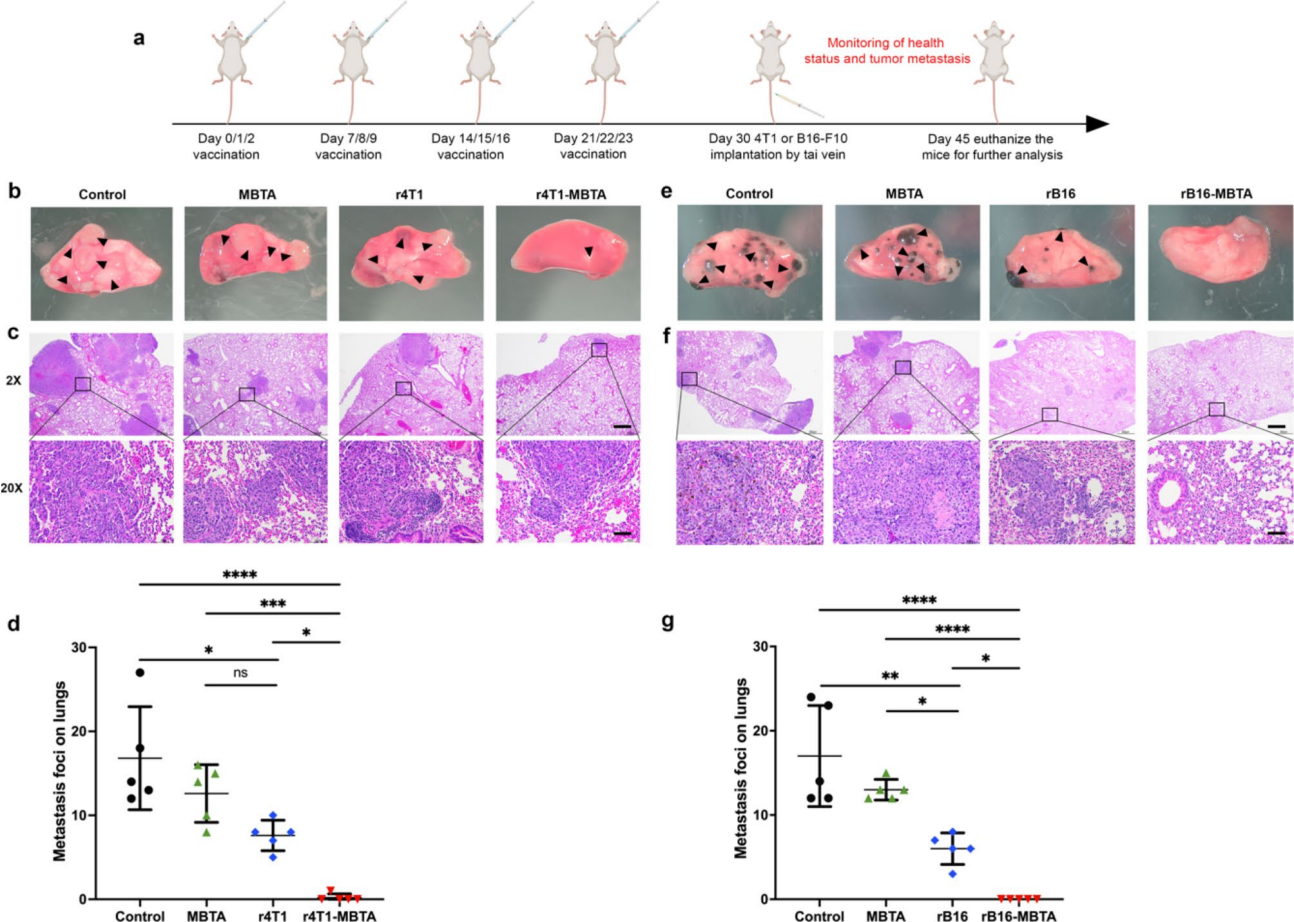
The rWTC-MBTA vaccine prevents tumor metastasis in breast and melanoma metastasis animal models. **a**, Treatment schedule for immunotherapy. **b&e**, Representative photographs of lung tissue from animal models of 4T1 or B16 metastasis. The black arrows indicate tumor metastasis foci (n = 5/group). **c&f**, Representative H&E staining of lung tissues collected on day 45 from (**c**) 4T1 or (**f**) B16 metastatic animal models (2× scale bar = 600 μm; 20 × scale bar = 60 μm) (n = 5/group). **d&g**, Quantification of metastatic foci in the lung mice using (**d**) 4T1 or (**g**) B16 metastasis model (n = 5/group). Ordinary one-way ANOVA with multiple comparisons was used to calculate statistical significance. *P < 0.05, **P < 0.01, ***P < 0.001,**** *P* < 0.0001.All data were represented as mean ± SD. *P*-values shown are for each treated group comparison

Building on our previous findings that rWTC-MBTA vaccination elicited a potent tumor-specific adaptive immune response and resulted in tumor regression in a colon cancer mouse model [13], we evaluated the vaccine’s ability to establish immunity against early tumor metastasis. To achieve this, we investigated the effect of r4T1-MBTA (irradiated 4T1 tumor cells mixed with MBTA) vaccination in a highly metastatic 4T1 breast cancer model by administering a tail vein challenge to 4T1 tumor cells. We conducted a standard 4-week r4T1-MBTA vaccination schedule and evaluated short-term immunity by 4T1 tail vein challenge one week after the completion of the vaccination schedule (Fig. 2a). Our results demonstrated that compared to the control, MBTA only, and r4T1 groups, the r4T1-MBTA vaccine significantly prevented lung metastasis (Fig. 2b-d and Supplementary Fig. 1a). We confirmed similar findings in a metastatic melanoma model, where rB16-MBTA vaccination led to a significant reduction in lung metastasis (Fig. 2e-g and Supplementary Fig. 1b).

To examine the potential of the rWTC-MBTA vaccine to generate long-term immunity, we challenged mice with tail vein 4T1 tumor cell implantation 90 days after the initial vaccination (Supplementary Fig. 2a). Our results showed that compared to the control group, the r4T1 group prevented a significant amount of metastasis to the lung. In contrast, the r4T1-MBTA vaccine resulted in a much more substantial suppression of metastasis (Supplementary Fig. 2b&c). These findings provide evidence that long-lasting immunity can be established by r4T1-MBTA vaccination and demonstrate its effectiveness as a prophylactic vaccine against early metastasis.

### rWTC-MBTA vaccine inhibits postoperative tumor metastasis in 4T1 breast cancer animal model

**Fig. 3:**
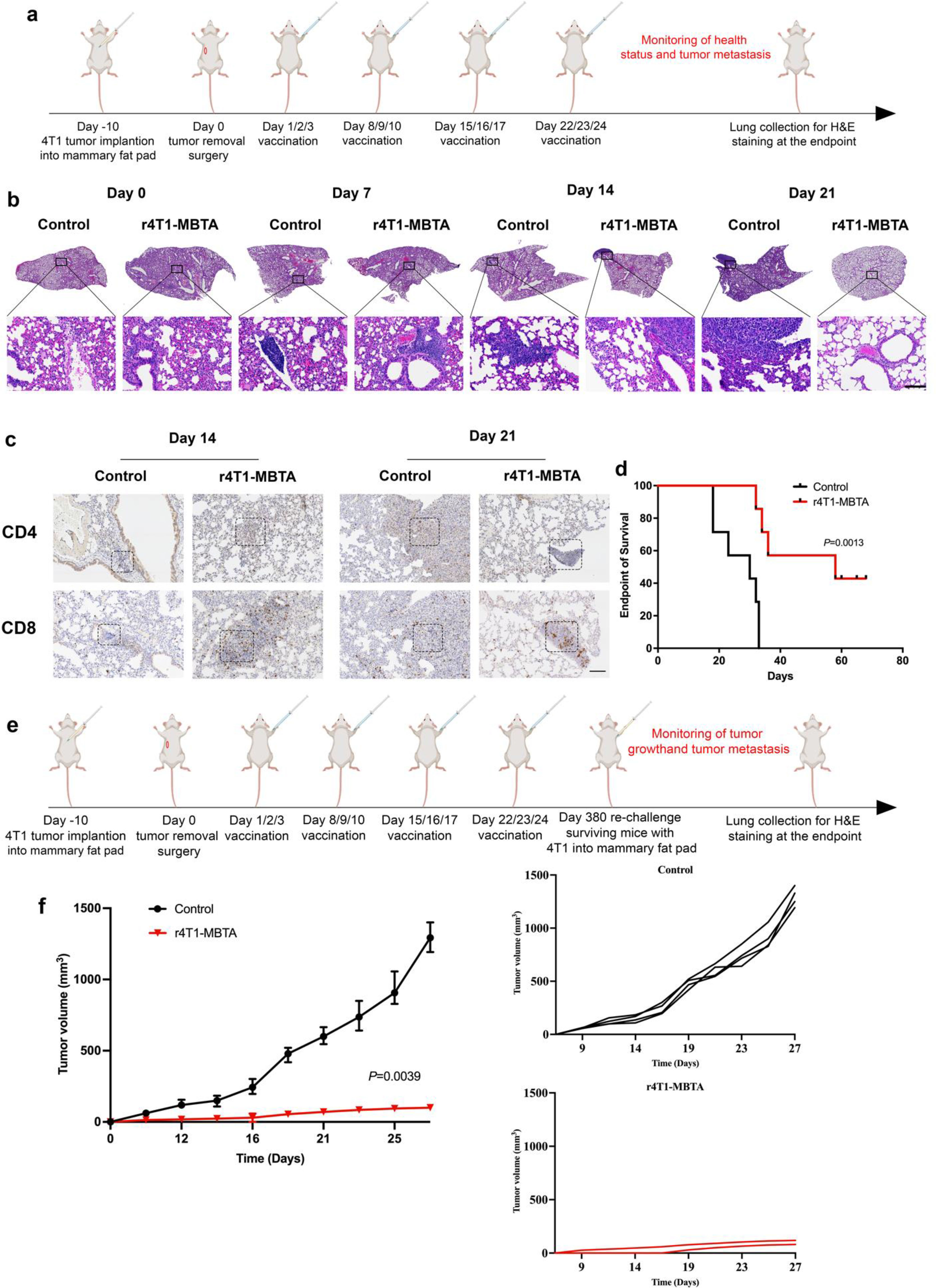
rWTC-MBTA vaccine inhibits postoperative tumor metastasis in 4T1 breast cancer animal model. **a**, Treatment schedule for immunotherapy in breast animal model with surgical tumor removal. **b**, Represantative H&E staining of lung tissue collected each at various time points. Scale bar = 100 μm (n = 3/group). **c**, IHC for CD4 and CD8 staining of lung tissue collected at different time points (n = 3/group). Scale bar = 100 μm. **d**, Overall survival curve of mice after control and vaccine treatments (n = 7/group). Survival rate was analyzed using the Mantel-Cox (log-rank) test. **e**, Treatment schedule for rechallenge with subcutaneous tumor cells in mice that survived the initial challenge. **f**, Mean and individual tumor growth curves for mice receiving different treatments (n = 4 for control, n = 2 for re-challenged survival mice). The mean tumor volumes were analyzed using Kruskal-Walllis test with multiple comparisons. All data were represented as mean ± SD. *P*-values are shown for control versus rWTC-MBTA vaccine

The robust antimetastatic efficacy observed with the rWTC-MBTA vaccine in our tail vein model led us to investigate whether this vaccine could prevent tumor metastasis in a more clinically relevant postsurgical model. We established orthotopic 4T1 tumor models by subcutaneously implanting 4T1 tumor cells into the 3rd mammary fat pad on the left side of BALB/c mice. Once the mean tumor volume reached 50 mm^3^ (approximately ten days after tumor implantation), the primary tumor was surgically removed (Fig. 3a). We then subcutaneously treated the resected mice over the right flank with PBS or r4T1-MBTA (Fig. 3a). To study the dynamic immune response process of tumor metastasis, we euthanized three mice per group each week after surgical resection. Upon histological analysis, no tumor was observed at the start or on day 0 in either the control or r4T1-MBTA vaccine group. However, on days 7 and 14, lung metastasis developed and grew in both groups. Multiple metastatic lesions were observed in the control group on day 21. In contrast, almost no metastatic lesions were observed in the r4T1-MBTA group (Fig. 3b & supplementary Fig. 3). Immunohistochemical staining of CD4 and CD8 in tumor samples from days 14 and day 21 showed increased CD4^+^ and CD8^+^ T cells in the r4T1-MBTA group (Fig. 3c). Consistently, the r4T1-MBTA vaccine significantly extended the median survival time from 30 days (control) to 58 days (rWT1-MBTA) (Fig. 3d).

We also investigated whether the r4T1-MBTA vaccine could induce long-term antitumor immune memory by rechallenging surviving mice with subcutaneous 4T1 cell implantation one year after the initial challenge (Fig. 3e). Tumors in rechallenged mice grew significantly slower than those in control mice (Fig. 3f). These results suggest that the r4T1-MBTA vaccine successfully generated potent antimetastatic and antitumor growth responses against breast tumors and induced long-term antitumor immune memory.

### The rWTC-MBTA vaccine impedes autologous tumor metastasis and growth but not allogeneic tumor metastasis and growth

**Fig. 4:**
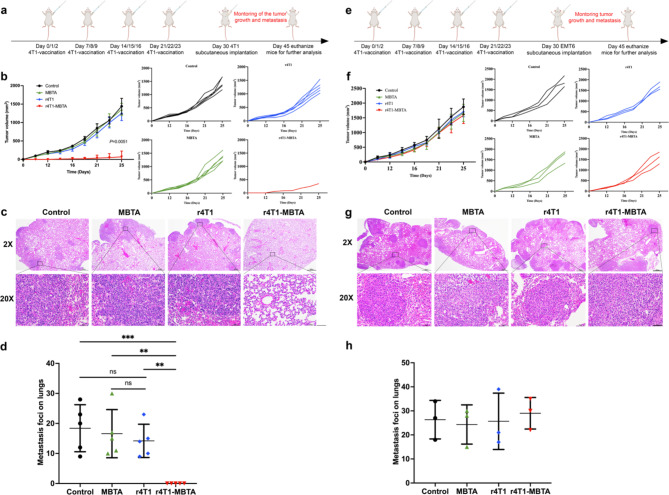
The rWTC-MBTA vaccine against autologous tumor metastasis and growth but not allogeneic tumor metastasis and growth. **a**, Treatment schedule for tumor growth and metastasis inhibition by the rWTC-MBTA vaccine. **b**, Mean and individual tumor growth curves for mice receiving different treatments (n = 5/group). The mean tumor volumes were analyzed using one-way ANOVA. **c**, Representative H&E staining of lung tissue collected at day 45 from the primary mammary animal model (2× scale bar = 600 μm; 20 × scale bar = 60 μm) (n = 3). **d**, Quantification of lung metastasis nodes in each group (n = 5/group). Ordinary one-way ANOVA with multiple comparisons was used to assess statistical significance. All data were represented as mean ± SD. *P*-values were shown for each treated group compared to rWTC-MBTA vaccine or r4T1. **e**, Cross-vaccination experiment design. **f**, Mean and individual tumor growth curves of the tumors receiving different treatments (n = 3/group). The data were presented as mean ± SD. **g**, Representative H&E staining of lung tissue collected on day 45 (2× scale bar = 600 μm; 20 × scale bar = 60 μm) (n = 3/group). **h**, Quantification of lung metastasis nodes in each group (n = 3/group). Ordinary one-way ANOVA with multiple comparisons was used to assess statistical significance. All data are represented as mean ± SD. *P*-values shown are for each treated group comparison. *P < 0.05, **P < 0.01, ***P < 0.001,**** *P* < 0.0001

Previous studies have demonstrated that autologous cancer vaccines present tumor-specific neoantigens more efficiently and generate antigen-specific immunological responses for personalized immunotherapy [23]. To verify the importance of incorporating autologous cancer cells into personalized tumor vaccines, we used two types of breast tumor cells (4T1 and EMT6) as autologous and allogeneic cell sources, respectively. Initially, all mice were immunized with r4T1-MBTA vaccines or control treatments (control, MBTA only and r4T1), and the treated mice were evenly divided into 4T1 and EMT6 groups. One week later, the treated mice were subcutaneously implanted with 4T1 or EMT6 tumor cells (Fig. 4a&e). In the subcutaneous tumor animal model, the r4T1-MBTA vaccine significantly inhibited 4T1 tumor growth (Fig. 4b) but did not affect EMT6 tumor growth (Fig. 4f). Furthermore, the r4T1-MBTA vaccine reduced lung metastasis by more than 95% compared with the control (Fig. 4c&d). Notably, r4T1 alone did not reduce lung metastasis to a significant extent compared to control alone. In EMT6 tumors, r4T1-MBTA and r4T1 was ineffective in preventing tumor metastasis compared to control (Fig. 4g&h). These findings underscore the critical role of autologous tumor cells in generating effective tumor vaccines and highlight their superiority over allogeneic sources for the personalization of cancer immunotherapy.

### rWTC-MBTA vaccine enhances antigen presentation by increasing the proportion of antigen-presenting cells

**Fig. 5:**
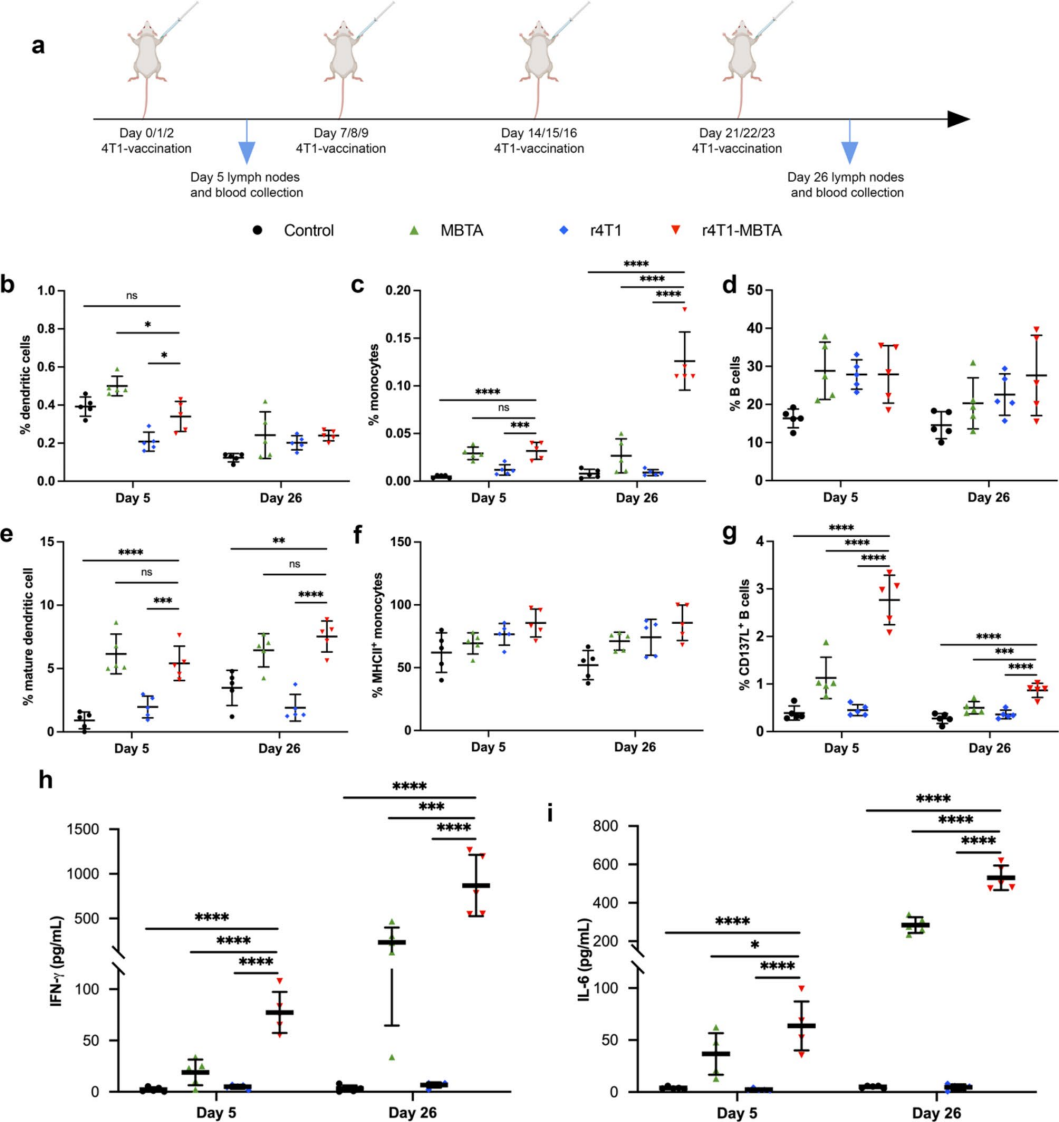
rWTC-MBTA vaccine enhances antigen presentation by increasing the proportion of antigen-presenting cells. After one or multi-dose treatments, lymph nodes (LNs) were collected to check the percentage of APCs by flow cytometry. **a**, Schematic diagram of the experimental design. **b**, Percentage of dendritic cells. **c**, Percentage of monocytes. **d**, Percentage of B cells. **e**, Percentage of mature DCs (gated as CD80^+^CD86^+^) after one or multi-dose of treatments. **f**, Percentage of MHCII^+^ monocytes in total monocytes after one or multi-dose treatments. **g**, CD137L^+^ B cells in total B cells after one or multi-dose treatments (gated on CD45 + cells). **h**, Serum concentrations of IFN-γ and **i**, IL-6 after one or multi-dose treatments. Ordinary one-way ANOVA with multiple comparisons was used to assess statistical significance. All data are represented as mean ± SD. *P*-values shown for each treated group compared to the rWTC-MBTA vaccine group (n = 5/group). *P < 0.05, **P < 0.01, ***P < 0.001, **** *P* < 0.0001

Antigen-presenting cells (APCs) are crucial for bridging the innate and adaptive immune response. Investigating their involvement in response to the rWTC-MBTA vaccine was prompted by their efficacy in preventing tumor metastasis and growth. We assessed the impact of the vaccine on APCs, including dendritic cells (DCs), monocytes, and B cells in the lymph nodes after different treatments in mice (Fig. 5a). We found that both the MBTA only and r4T1-MBTA vaccine triggered innate immune responses shortly after administration. The percentage of dendritic cells (DCs) (Fig. 5b), monocytes (Fig. 5c), and B cells (Fig. 5d) within the total CD45^+^ cell population significantly increased compared to the control group. This increase confirms the potent stimulating effects of Mannan, TLR ligands, and CD40 antibody on innate immune cells. Compared with the control group, the percentage of DCs in the rWTC-MBTA vaccine increased 1.9-fold on day 26, the percentage of monocytes increased 6.4-fold on day 5 and more than 10-fold on day 26, and the percentage of B cells rose 1.7-fold on day 5 and 1.9-fold on day 26. Moreover, the r4T1-MBTA vaccine markedly induced the maturation of DCs (CD80^+^CD86^+^) (Fig. 5e), MHCII^+^ monocytes (Fig. 5f), and CD137L^+^ B cells (Fig. 5g) on day 5 and day 26, confirming that r4T1-MBTA activated APCs. The r4T1-MBTA vaccine induced a 6-fold DC maturation percentage on day 5 and 2.2-fold on day 26, increased 1.4-fold MHCII + monocyte percentage on day five and 1.6-fold on day 26, and induced a 7.1-fold CD137L^+^ B cell percentage on day 5 and 3.2-fold on day 26 compared to the control group.

We analyzed vaccine-induced stimulation of pro-inflammatory cytokines interleukin 6 (IL-6) and interferon-gamma (IFN-γ) via ELISA. Our results showed that the r4T1-MBTA vaccine significantly promoted the secretion of IFN-γ and IL-6 compared to the control, MBTA only, or r4T1 groups (Fig. 5h&i). The serum levels of IFN-γ and IL-6 in the r4T1-MBTA vaccine group were 4-fold and 1.7-fold higher than those in the MBTA group, respectively, and 15.5-fold and 26.9-fold more elevated than those of the r4T1 group examined on day 5 (3 days post first vaccination). Furthermore, the levels of IFN-γ and IL-6 in the r4T1-MBTA group were 3.7-fold and 1.9-fold higher than those in the MBTA group, respectively, and 131-fold and 113.6-fold higher than those of the r4T1 group examined on day 26 (3 days after the last vaccination). These findings confirmed that the r4T1-MBTA vaccine significantly stimulated antitumor innate immunity through APC induction and elicited a pro-inflammatory immune response in-vivo.

### The rWTC-MBTA vaccine stimulates anti-metastasis adaptive immunity

**Fig. 6:**
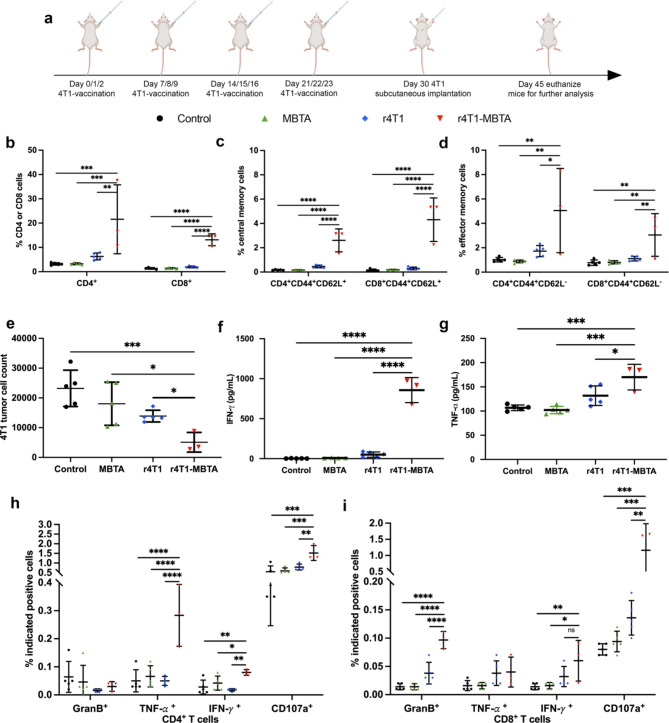
The rWTC-MBTA vaccine stimulates anti-metastasis adaptive immunity. Spleens were collected and isolated at the endpoint of the 4T1 subcutaneous metastasis animal model (**a**). CD4^+^ and CD8^+^ T cells were examined by flow cytometry in splenocytes from different treatment groups. **b**, Percentage of T-cells (CD4 ^+^ or CD8^+^) in splenocytes from different groups. **c**, Percentage of central memory (CD44^+^CD62L^+^) T cells in splenocytes of different treatment groups. **d**, Percentage of effector memory (CD44^+^CD62L^−^) CD4 and CD8 T cells in splenocytes. **e-i**, All the analyses are based on the co-culture 4T1 tumor cells and indicated splenocytes from different treated animals. **e**, 4T1 tumor cell number count after co-culture. **f & g**, Concentrations of cytokines IFN-𝛾 (**f**) and TNF-𝛼 (**g**) from co-culture supernatants as measured by ELISA. **h&i**, Percentage of Gran B^+^, IFN-𝛾^+^, TNF-𝛼^+^, or CD107^+^ CD4^+^ (**h**) or CD8^+^ T cells (**i**) was determined by flow cytometry. The analysis from **e** to **i** was conducted after co-culturing splenocytes (from different treated mice) with 4T1 tumor cells. Ordinary one-way ANOVA with multiple was used to assess statistical significance. All data are represented as mean ± SD. *P*-values are shown for each treated group compared to the rWTC-MBTA vaccine group (n≥3/group). *P < 0.05, **P < 0.01, ***P < 0.001, **** *P* < 0.0001

To further investigate the mechanisms responsible for the enhanced antitumor efficacy of r4T1-MBTA vaccination, we conducted flow cytometry-based immunophenotyping to evaluate the populations of memory and cytotoxic T cells (Fig. 6a). Analysis of T cell-mediated immunity demonstrated that the r4T1-MBTA vaccine elicited a significantly higher proportion of CD4^+^ and CD8^+^ T cells in the spleen (Fig. 6b). In the r4T1-MBTA vaccine group, the CD45^+^CD4^+^ cell population increased 6-fold. The CD45^+^CD8^+^ population increased by 5-fold compared to the control group. Within these T-cell subsets, the r4T1-MBTA vaccine group exhibited a 5-fold higher percentage of effector memory (CD44^+^CD62L^−^) and a 15-fold increase in the proportion of central memory (CD44^+^CD62L^+^) proportion in CD4^+^ T-cells. In contrast, CD8^+^ T-cells showed a 4-fold increase in effector memory and a 26-fold increase in central memory cells (Fig. 5c&d).

Co-culturing of splenocytes confirmed the T-cell-mediated cytotoxicity in 4T1 tumor cells from each group. The data indicated a 78% increase in 4T1 tumor cell death in r4T1-MBTA splenocytes compared to controls and a 72% and 63% increase compared to MBTA-only and r4T1 splenocytes, respectively (Fig. 6e). We also evaluated the cytokine expression in the co-culture system using ELISA and flow cytometry. The ELISA results showed that the r4T1-MBTA vaccine significantly promoted the secretion of interferon-gamma (IFN-γ) and tumor necrosis factor (TNF-α). For instance, the IFN-γ levels in the co-cultured supernatant from the r4T1-MBTA vaccine group were 92-fold and 16.8-fold higher than those of the MBTA-only and r4T1 groups, respectively (Fig. 6f). Flow cytometry revealed that the r4T1-MBTA vaccine increased TNF-α^+^, IFN-γ^+^, and CD107a ^+^ CD4^+^ T-cells and GranB^+^, IFN-γ^+^, and CD107a^+^ CD8^+^ T cells. The percentage of TNF-α^+^, IFN-γ^+^, and CD107a^+^ positive CD4^+^-T cells in the rWTC-MBTA vaccine group increased by 5.7-fold, 2.9-fold, and 2.8-fold, respectively, compared to the control group (Fig. 6h). The percentage of cells positive for Granzyme B (GranB), IFN-γ, and CD107a expression increased 6.9-fold, 4.3-fold, and 14.5-fold, respectively, in CD8 ^+^ T-cells in r4T1-MBTA mice compared to control mice (Fig. 6i).

To validate whether the rWTC-MBTA vaccine could induce T-cell activation in a syngeneic melanoma animal model with B16-F10 cells, we immunized C57BL/6 mice with the rB16-MBTA vaccine according to the treatment schedule in Supplementary Fig. 4a. Results showed that the rB16-MBTA vaccine was as effective as the r4T1-MBTA vaccine in enhancing T-cell activation and cytotoxicity (Supplementary Fig. 4). These findings confirmed that the rWTC-MBTA vaccine could significantly induce adaptive antitumor immunity, thereby preventing tumor growth and metastasis.

### rWTC-MBTA vaccine against early tumor metastasis is T-cell dependent

**Fig. 7:**
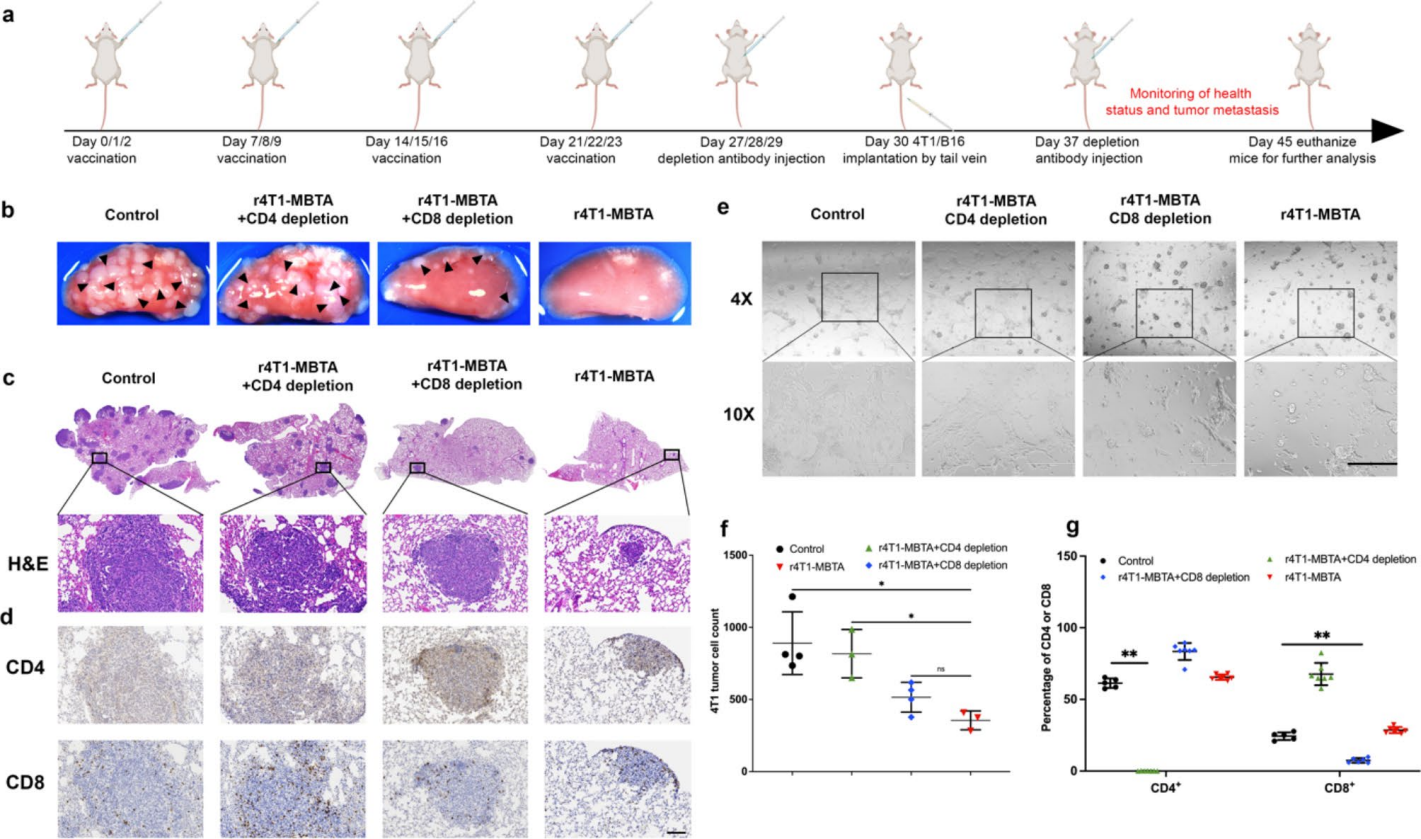
rWTC-MBTA vaccine against early tumor metastasis depends on T cells. 4 weeks post-vaccination were treated with three doses of anti-CD8α or anti-CD4 antibody (250 µg/mouse) as shown in the schematic diagram (**a**). **b&c** Representative photographs and H&E staining of lung tissue from different treatment groups. **d**, Representative IHC staining of lung tissues for CD4 and CD8 markers from different treatment groups (scale bar = 100 μm). **e**, Representative photographs 48 h after co-culture of purified T cells from splenocytes and 4T1 tumor cells (scale bar = 400 μm). **f**, Tumor cell counting by flow cytometry 48 h after co-culture of T-cell and 4T1 tumor cells. Ordinary one-way ANOVA with multiple comparisons was used to assess statistical significance. All data are represented as mean ± SD. *P*-values are shown for each treated group compared to the rWTC-MBTA vaccine group. **g**, CD4^+^ T cells and CD8^+^ T cells from splenocytes from different treatments were detected via flow cytometry. Mann-Whitney test was used to assess statistical significance. All data were represented as mean ± SD (n≥3/group). *P < 0.05, **P < 0.01, ***P < 0.001, **** *P* < 0.0001

The r4T1-MBTA vaccine has been observed to induce antitumor and antimetastatic immunity in syngeneic mice, indicating the potential involvement of the adaptive immune system. To confirm this, we depleted CD4 or CD8 T cells in vaccinated mice (Fig. 7a) and verified the depletion efficiency via flow cytometry, which demonstrated almost 99% CD4 depletion and 70% CD8 depletion with the respective antibodies (Fig. 7g). As expected, the lack of either CD4 or CD8 T cells failed to elicit an antitumor immune response compared to control mice, resulting in the development of lung metastases following the injection of 4T1 tumor cells via the tail vein (Fig. 7b&c and supplementary Fig. 5a). In CD4-depleted mice, the number of metastatic lung loci increased by about 23-fold. In contrast, in CD8-depleted mice, it increased by approximately 10-fold compared to r4T1-MBTA vaccinated mice. These findings suggest that T cells, particularly CD4 T cells, are critical for the antimetastatic efficacy of vaccines. Immunohistochemical staining of CD4 and CD8 in the lung tissues of each group further confirmed significant infiltration of CD4^+^ and CD8^+^ T cells in the r4T1-MBTA vaccine group, with fewer CD4^+^ and CD8^+^ T cells observed in the CD4 and CD8 depletion groups, respectively (Fig. 7d). To verify T cell-mediated cytotoxicity, we co-cultured purified T cells from each group with 4T1 or EMT6 tumor cells. T-cells from the r4T1-MBTA vaccine group showed significant cytotoxicity against 4T1 cells (over 60% 4T1 tumor cell death compared to the control), with partial cytotoxicity observed in splenocytes from the CD8-depleted r4T1-MBTA vaccinated group (around 42% 4T1 tumor cell death compared to the control) (Fig. 7e&f and supplementary Fig. 5b). However, splenocytes from CD4-depleted mice only killed 8% of the 4T1 tumor cells (Fig. 7e&f), consistent with the histological results (Fig. 7b&c). Notably, splenocytes from all four groups did not exhibit cytotoxicity against EMT6 tumor cells, indicating T-cell specificity retention (supplementary Fig. 6) consistent with previous results (Fig. 4f, g&h). These results confirmed that the efficacy of the r4T1-MBTA vaccine against autologous tumor metastasis is T-cell dependent, with CD4 T cells playing a predominant role.

### r4T1-MBTA vaccine toxicity evaluation

**Fig. 8:**
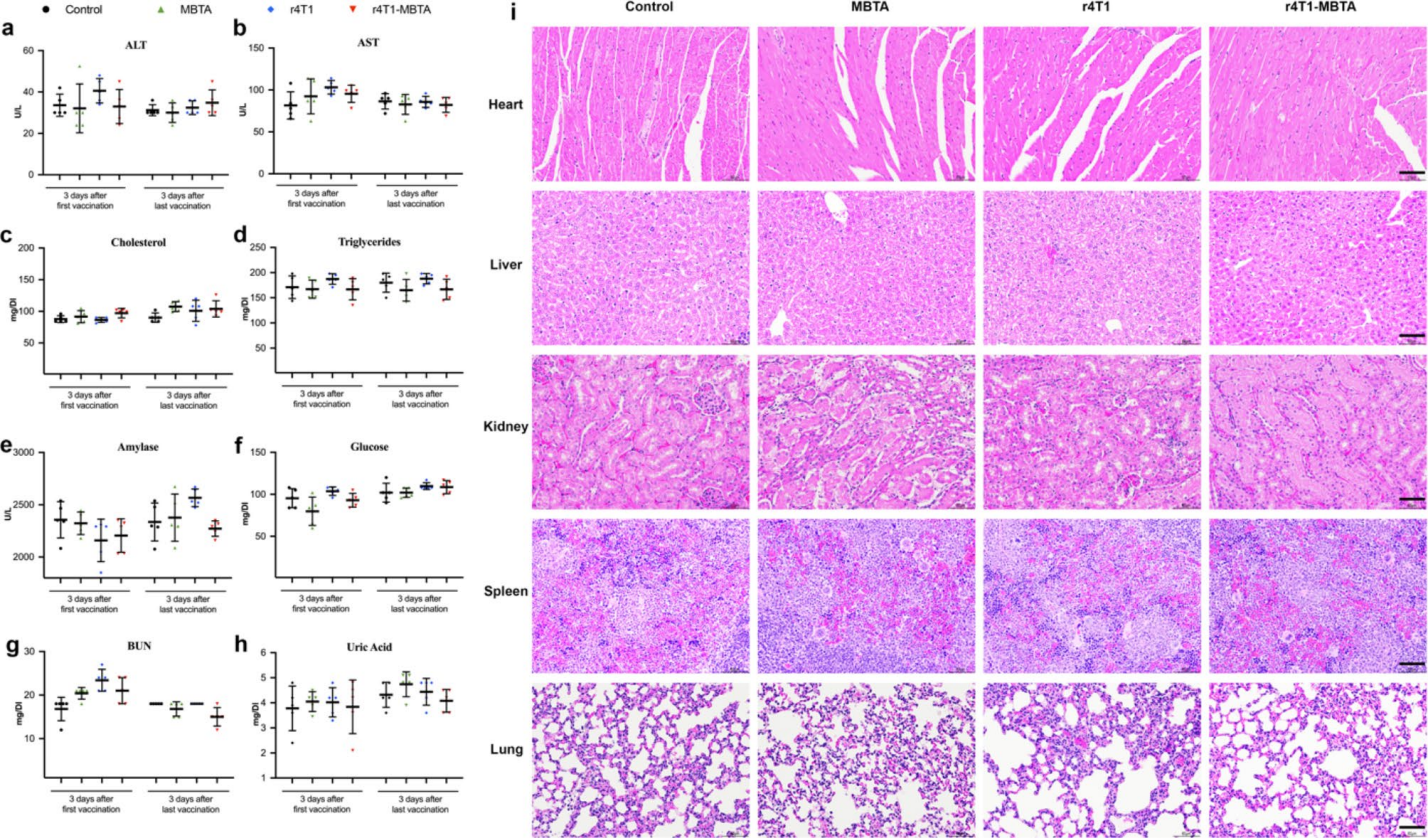
r4T1-MBTA vaccine toxicity evaluation. Serum levels of **a**, ALT; **b** AST; **c** cholesterol; **d**, triglycerides; **e**, amylase; **f**, glucose; **g**, BUN and **h**, uric acid after 1 or 12 dose injections with different treatments. All parameter values are in the normal range and there was no significant difference in either treatment (MBTA, IR only, and rWTC-MBTA vaccine) group compared with the control group using one-way ANOVA with multiple comparisons. **i**, Representative H&E staining of major organs after multiple dose injections. Scale bar = 60 μm. All data were represented as mean ± SD (n = 5/group)

Experimental immunotherapies are often limited by systematic toxicity. To assess the potential toxicity of the r4T1-MBTA vaccine, we conducted a thorough evaluation using routine complete blood counts and biochemical indexes on mice that received different treatments. The results showed no significant differences in biochemical indexes including aspartate aminotransferase (AST), alanine aminotransferase (ALT), cholesterol, triglycerides, amylase, glucose, blood urea nitrogen (BUN), and uric acid, between the treatment and control groups either three days after the first dose (3 injections) or three days after the last dose (12 injections) (Fig. 8a-h. These findings suggest that the vaccine did not cause hepatotoxicity or nephrotoxicity. Additionally, no pathological changes in the major organs (i.e., heart, liver, spleen, lung, and kidney) were observed in any of the treatment groups compared to the control group, as confirmed by H&E staining (Fig. 8i). Based on these hematological and histopathological analyses, we concluded that the r4T1-MBTA vaccine is highly biocompatible and can serve as a safe and effective therapeutic option.

## Discussion

Despite recent advances in cancer therapeutics, including immunotherapy, metastasis remains the primary and foremost reason for cancer-related fatalities [24]. Metastasis is a complex and multifaceted process and is one of the major obstacles against effective cancer treatment, as cancer cells leave the primary tumor site and colonize distant organs through the nefarious “metastatic cascade” [25]. Though conventional therapies like surgical removal of tumor mass, radiation therapy, and chemotherapy can cure the primary tumor, metastatic tumors often resist traditional therapies and occur in tandem with poor prognosis and a higher death rate [26]. Target therapies and immunotherapies are the contemporary centerpieces in treating and preventing metastatic cancers [25]. In recent years, immunotherapy has shown significant promise for treating several recurrent or metastatic tumors. However, immunotherapeutic approaches like CAR-T and checkpoint inhibitors have not demonstrated considerable aptitude in preventing cancer metastasis [27, 28]. Autologous tumor cell-based vaccines are a relatively new front in cancer immunotherapy and have yet to yield definitive efficacy against solid tumors [10].

In the field of tumor vaccine therapy, researchers are faced with some major challenges that need to be addressed: improving the weak immunogenicity of tumor-derived antigens, impaired antigen presentation by the APCs, countering the immune escape mechanism of tumors and an immune-suppressive tumor microenvironment, and achieving effective delivery of tumor vaccines [29]. To overcome these obstacles, we propose an autologous tumor cell vaccine (rWTC-MBTA), which can exert robust antitumor immune responses between the critical window of growth of primary tumors and the onset of metastatic tumors. We demonstrate that vaccination with rWTC-MBTA inhibited tumor metastasis and recurrence in several preclinical mice models of metastatic tumors. This study also showed the generation of systemic antitumor immunity that eliminates the existing spontaneous metastasis. To mimic the clinical scenario, we used a postoperative orthotopic 4T1 tumor model, where the primary tumor is removed surgically, and even in this model, rWTC-MBTA effectively cured the metastatic disease. Altogether, these data validate rWTC-MBTA’s capability to inhibit tumor metastasis, offering a promising resolution for preventing and treating metastatic disease.

In our previous study we focused solely on evaluating the therapeutic efficacy of the rWTC-MBTA vaccine, with limited exploration of the underlying mechanisms or its efficacy in preventing tumor metastasis [13]. However, recognizing the need for preventive vaccines, our current study investigated the preventive efficacy of the rWTC-MBTA vaccine in multiple metastasis animal models and shed light on its anti-metastatic mechanism.

As shown in our previous study, our vaccine is composed of irradiated whole tumor cells (rWTC) labeled with mannan-BAM, Toll-like receptor (TLR) ligands, and an anti-CD40 antibody [13]. Briefly, mannan, a pathogen-associated molecular pattern (PAMP), binds to pattern recognition receptors (PRRs) on innate immune cells such as dendritic cells and macrophages, promoting opsonization and cytokine release, including TNF-α and IL-6 [30, 31]. TLR agonists, including resiquimod, polyinosinic-polycytidylic acid, and lipoteichoic acid, act as adjuvants by binding to specific TLRs on innate immune cells, initiating proinflammatory signaling cascades and polarizing the immune response [32, 33]. The anti-CD40 antibody interacts with the CD40 receptor on dendritic cells, activating them and improving antigen processing and presentation to T cells [34]. While our previous study highlighted the therapeutic efficacy of the rWTC-MBTA in a mouse colon cancer model, the underlying mechanism was not fully explored. In our current study, we aim to investigate its preventive efficacy against tumor metastasis by conducting experiments in multiple metastasis animal models. We also sought to unravel the intricate mechanisms underlying its anti-metastatic effects, thus paving the way for potential preventive strategies in cancer metastatic treatment.

As demonstrated by our results, both the MBTA-only and rWTC-MBTA groups triggered innate immune responses shortly after administration. These responses were characterized by increased dendritic cells, mature DCs (CD80^+^CD86^+)^, monocytes in the lymph nodes, and pro-inflammatory cytokines IFN-γ and IL-6 in the serum (Fig. 5). This is likely due to the presence of Mannan, TLR ligands and CD40 antibody which are known to be strong stimulators of innate immune cells. However, tumor-specific cytotoxicity was only observed in the rWTC-MBTA treatment group but not in the MBTA-only group, likely due to lack of a source for tumor antigens. This is further evidenced by our co-culture experiments, in which only splenocytes from the rWTC-MBTA group efficiently killed tumor cells and secreted the highest level of IFN-γ (Fig. 6e&f). Furthermore, our analysis of immune cell subsets revealed that the rWTC-MBTA group exhibited increased pan-, effector memory-, and central memory- CD4 + and CD8 + T cells (Fig. 6). Previous studies have shown that these cells are vital to controlling tumor metastasis [35, 36]. Patients with advanced metastatic disease often develop a recurrent tumor at distant tissues even after surgical removal of the primary tumor. It is important to consider the possibility of disseminated cancer cells even in the cases of early-stage cancer where the primary tumor is completely removed through surgery, but the patient experiences a relapse with metastatic disease. This indicates that cancer cells may have already spread to distant organs before the tumor was removed. For patients who have had their cancer surgically removed but remain at risk of later metastatic relapse, adjuvant and/or neoadjuvant therapy activating innate and adaptive immunity has proven to be effective in reducing recurrence rates and prolonging overall survival [23, 37]. However, rWTC-MBTA vaccine is a step-up over the adjuvant and neoadjuvant therapy, using autologous tumor cells in the preparation as a source of tumor specific antigens.

The disconnect between preclinical studies and first-in-human clinical trials has led to several therapeutics that show promise in controlling micrometastatic tumor but failing to achieve shrinkage of metastatic tumors in human clinical trials. As a result, drugs that could potentially control micrometastatic disease often don’t reach the patients who would benefit the most. Our postoperative breast cancer metastasis model aimed to replicate this clinical scenario and evaluates the potential of the rWTC-MBTA vaccine as a strategy for early intervention against metastatic disease. Our results suggest that the rWTC-MBTA vaccine has the potential to disrupt the progression of lung metastasis, as evidenced by the histological examination of lung tissues at various time points after surgical removal of the primary tumor (Fig. 3). On day 0 following tumor removal, no visible tumor was observed. On day 7 and day 14, both the control and vaccinated mice exhibited small tumor nodules. However, by day 21, the tumor nodules in the control group had increased in size, while those in the vaccinated group had significantly reduced. (Fig. 3b& supplementary Fig. 3). Additionally, via immunohistochemistry the vaccinated mice showed a significant increase in the infiltration of CD4 and CD8 T cells in the metastatic tumors (Fig. 3c). These findings suggest that the rWTC-MBTA vaccine holds promise as a strategy for early intervention to prevent and treat metastatic breast cancer, addressing the unresolved challenge of preventing metastatic seeding and colonization of tumors. Furthermore, our T-cell depletion data showed that the efficacy of r4T1-MBTA vaccine’s efficacy against autologous tumor metastasis is T-cell dependent, with CD4 T cells playing a predominant role (Fig. 7). This finding is consistent with other studies that have shown the importance of CD4 T cells in mounting an effective antitumor immune response and preventing tumor metastasis [38, 39].

By targeting tumor-associated antigens (TAAs), which are proteins that are aberrantly expressed by metastatic cancer cells, therapeutic vaccines like rWTC-MBTA have the potential to stimulate broad-based anti-tumor immunity. Furthermore, neoantigens, which are specific to tumor cells and arise from sporadic non-synonymous mutations, can be targeted by therapeutic vaccines to specifically and effectively direct the immune system to eliminate cancer cells. Tumor-specific T-cell response can be triggered by neoantigens, which are only expressed by tumor cells. This approach is effective in minimizing “off-target” damage, making it a promising strategy in cancer treatment. Notably, irradiated tumor cells alone induced a favorable adaptive immune response, including a slight increase in pan-, effector memory-, and central memory- CD4^+^ and CD8^+^ T cells, which was significantly amplified with rWTC-MBTA (Fig. 6b-d). These findings align with our previous data, which showed that the rCT26-MBTA vaccine significantly enhanced CD8^+^ T cells in the blood and tumor, while rCT26 alone had only a marginal effect [13]. This is also consistent with previous studies indicating that irradiated tumor cells serve as a neoantigen pool for tumor antigen presentation [40]. The adjuvants in the rWTC-MBTA strategy significantly enhanced neoantigen presentation and tumor-specific T-cell activation [41]. Without a neoantigen pool from irradiated tumor cells, MBTA alone could not elicit tumor-specific adaptive immune responses and failed to prevent or treat tumor metastasis. Overall, the rWTC-MBTA vaccine with a neoantigen pool holds promise as a targeted and personalized approach for cancer immunotherapy. A limitation of this study is that we did not elucidate the effects of neo-antigen presentation on T-cell polyclonality. Future work will be needed to analyze the t-cell repertoire in response to rWTC-MBTA vaccine and could shed insight into the benefits of autologous tumor vaccines. Keeping this in mind, our rWTC-MBTA vaccine employs mannan-BAM as a measure to increase the immunogenicity of cancer cells. Mannan-BAM augments the immunogenicity of the cancer cells and concomitant antigen presentation by APCs by facilitating opsonization and phagocytosis [29]. This approach is not employed by any other autologous whole cell cancer vaccines previously and clearly adds an edge over other cancer vaccines in targeting the sporadic neoantigens of metastatic cancers [42]. It is commonly observed that metastatic human cancers tend to have a higher mutational burden and are more aneuploid as compared to genetically engineered mouse tumors. However, it is still not very clear to what extent even the most complex mouse models can mimic the neoantigen landscape and immune microenvironment of advanced metastatic cancer in human patients [43, 44]. Now, there are no definite conclusions on this topic. Nevertheless, the development of humanized mice has paved the way for patient-specific tumor organoid and immune cell reconstitution in an orthotopic in vivo environment, which is a significant step forward in this field [45].

Furthermore, cytokine release syndrome (CRS) is a potentially serious condition characterized by an excessive immune response triggered by certain immunotherapies, particularly those that utilize T-cells [46]. CRS can manifest as a collection of symptoms ranging from mild to severe, including fever, chills, fatigue, headache, muscle aches, nausea, vomiting, diarrhea, low blood pressure, rapid heartbeat, and in severe cases, organ dysfunction or failure [47]. In our previous study the biosafety profile of our vaccine was not thoroughly elucidated [13]. In this study, we thoroughly monitored the mice for CRS in relation to our rWTC-MBTA vaccine group. Blood samples were collected at different time points to assess hematological (related to serum) and histopathological (related to tissue structure) parameters. These analyses aimed to determine if our rWTC-MBTA may trigger CRS as a potential side effect. Based on the toxicity data obtained from our analyses, our rWTC-MBTA demonstrated no signs of CRS-related hematological or histopathological abnormalities (Fig. 8). These findings provide further evidence of the safety profile of our rWTC-MBTA in relation to the potential risk of CRS associated with immunotherapy treatments involving T-cells. All the data indicated that rWTC-MBTA could induce an antitumor immune response against early metastasis after surgical removal of primary tumors, providing a potential strategy for early intervention against breast cancer and melanoma metastasis.

The rWTC-MBTA vaccine has a major advantage in that it can impact the immunogenic status of the tumor microenvironment (TME), which can help to achieve better outcomes. One of the biggest challenges in cancer immunotherapy is the “cold” TME of some solid tumors. This is because the immunogenically cold TME is associated with lower neoantigen presentation and a lack of inflammatory T-cell infiltration [48]. However, TLR agonists in the rWTC-MBTA preparation can promote Th1-mediated inflammatory responses and activate APCs in TME, which can lead to better tumor infiltration and improved functioning of the immune effector cells, like NK cells and CD8^+^ T cells. The Th1 inflammatory cytokines polarize and increases CD8 + T effector function and impedes Treg cell activity. By manipulating the TME, the MBTA vaccine approach with the use of TLR agonists can turn the “cold” TME “hot,” it has been effective in curbing immunogenically cold and highly metastatic tumors like triple-negative breast cancer, as depicted by our results. A mutualistic therapeutic combination of rWTC-MBTA with ICIs, can lead to a practical approach to treating metastatic cancers that were previously immune-unresponsive, and is currently being validated in further research projects in our laboratory.

## Conclusion

In conclusion, our study provides evidence of the potential of the rWTC-MBTA vaccine as a promising approach for preventing and treating metastatic cancers. However, further investigations are warranted to fully evaluate its safety and determine the optimal dosing and treatment duration before translation to the clinical setting. In the future, we envision a process where the rWTC-MBTA vaccine could be prepared from resected tumors and administered back to patients with multiple malignancies, potentially improving the quality of life.

## Electronic supplementary material

Below is the link to the electronic supplementary material.

Supplementary Material 1

Supplementary Material 2

Supplementary Material 3

## Data Availability

Data supporting the results of this study can be found in the article and its supplements. The reagents used in this study are available from the corresponding authors upon request.

## Abbreviations

rWTC-MBTA: Irradiation of whole tumor cell-Mannan BAM plus with toll receptor agonists and anti-CD40
ATVs: Autologous tumor cell-based vaccines
BAM: Biocompatibility Anchors for Cell Membranes
MB: Mannan-BAM
PAMP: Pathogen-associated molecular pattern
TA: TLR agonists and anti-CD40 antibodies
MBTA: Mannan-BAM, TLR agonists, and anti-CD40 antibody
FBS: Fetal bovine serum
APCs: Antigen-presenting cells
DCs: Dendritic cells
IFN-γ: Interferon-γ
IL-6: Interleukin 6
TNF-α: Tumor necrosis factor
GranB: Granzyme B
ALT: Serum alanine aminotransferase
AST: Aspartate transaminase
BUN: Blood urea nitrogen
R-848: Resiquimod 848
poly(I:C): Polyinosinic-polycytidylic acid
LTA: Lipoteichoic acid
PRRs: Pattern recognition receptors
ACUC: Animal Care and Use Committee
NHS: N-hydroxysuccinimide
SD: Standard deviation
CRS: Cytokine Release Syndrome
