# Supplementary material for: rWTC-MBTA: autologous vaccine prevents metastases via antitumor immune responses
Source: J Exp Clin Cancer Res. 2023 Jul 12;42:163. doi: 10.1186/s13046-023-02744-8 (PMC10337177; doi:10.1186/s13046-023-02744-8)
Supplement: Supplementary file 1 — Supplementary Material 1 [file 13046_2023_2744_MOESM1_ESM.docx]

Supplementary figures and legends:

rWTC-MBTA: Autologous Vaccine Prevents Metastasis via Antitumor Immune Responses

Juan Ye^1#^, Herui Wang^1#^, Rogelio Medina^1^, Samik Chakraborty^2^, Mitchell Sun^1^, Alex Valenzuela^1,3,4^, Xueyu Sang^1^, Yaping Zhang^1^, Ondrej Uher^5^, Jan Zenka^6^, Karel Pacak^5^, Zhengping Zhuang*^1^

1, Neuro-Oncology Branch, National Cancer Institute, National Institutes of Health, Bethesda, Maryland, USA.

2, NE1 Inc., New York, NY.

3, David Geffen School of Medicine, University of California, Los Angeles, CA, USA;

4, Charles R. Drew University of Medicine and Science, Los Angeles, CA, USA

5, Section on Medical Neuroendocrinology, Eunice Kennedy Shriver National Institute of Child Health and Human Development, National Institutes of Health, Bethesda, Maryland, USA

6, Department of Medical Biology, Faculty of Science, University of South Bohemia, České Budějovice, Czech Republic

# These authors contributed equally

*Correspondence:

Zhengping Zhuang, M.D., Ph.D.

Senior Investigator

Neuro-Oncology Branch

National Cancer Institute

Center for Cancer Research

National Institutes of Health

Building 37 Room 1000

37 Convent Dr.

Bethesda, MD 20892

zhengping.zhuang@nih.gov


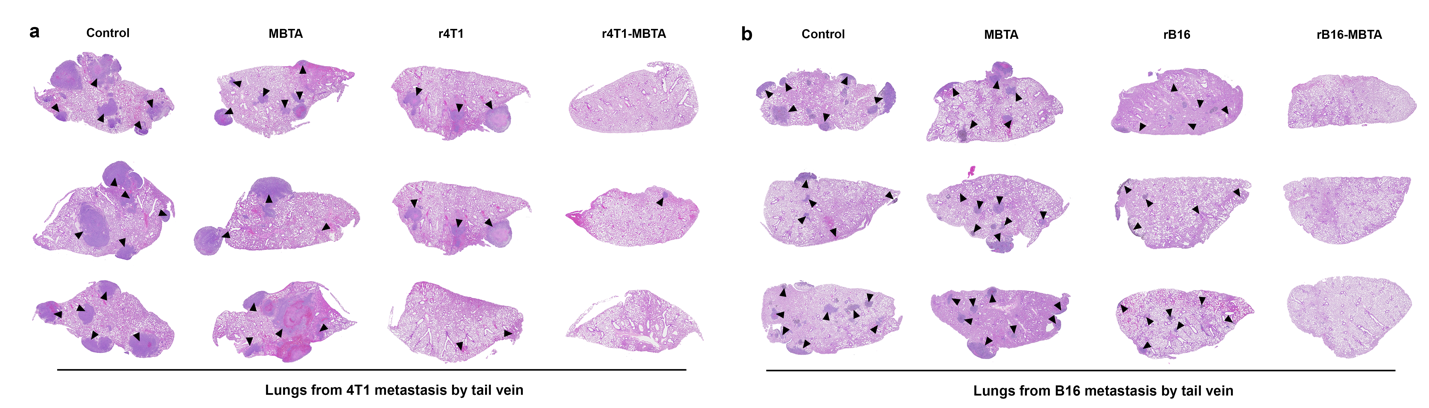


**Supplementary Figure 1. H&E staining of lung sections collected from 4T1 or B16 metastasis by tail vein animal model.** (Corresponding to Figure 2**)** Representative H&E staining of the lung tissue collected at day 45 from 4T1 (**a**) and B16 (**b**) metastasis animal model. The black arrows indicate tumor metastasis focus (n=3/group).


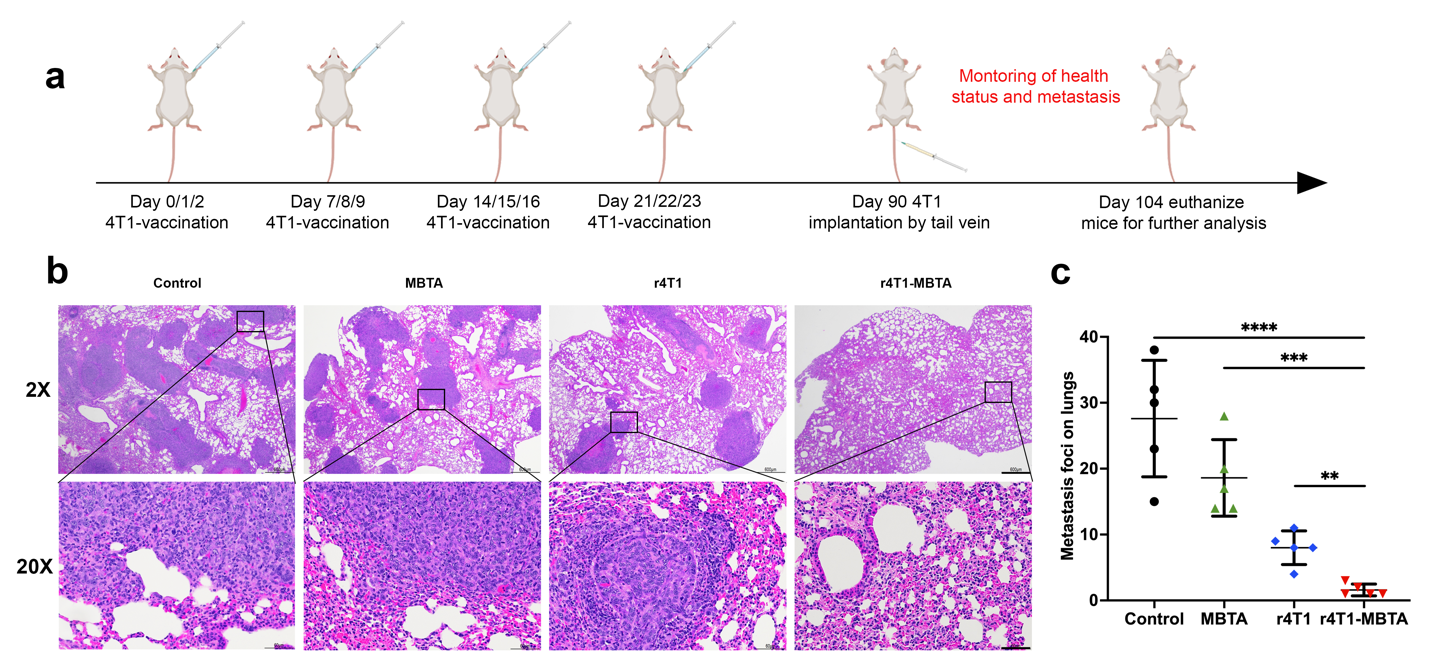


**Supplementary figure 2. Metastasis prevention via rWTC-MBTA vaccine induced long-term immune memory effects. a,** Therapeutic schedule for rWTC-MBTA vaccine-mediated inhibition of tumor metastasis. **b,** Representative H&E staining of the lung tissue collected at day 104 (2× scale bar = 600 μm; 20 × scale bar = 60 μm). **c,** Quantification of lung metastasis nodes in each group. Ordinary one-way ANOVA with multiple comparisons was used to assess statistical significance. All data are represented as mean ± SD. *P*-values are shown for each treated group (control, MBTA only, or rWTC only group) versus the rWTC-MBTA vaccine (n=5/group). **P*<0.05, ***P*<0.01, ****P*<0.001,**** *P*<0.0001.



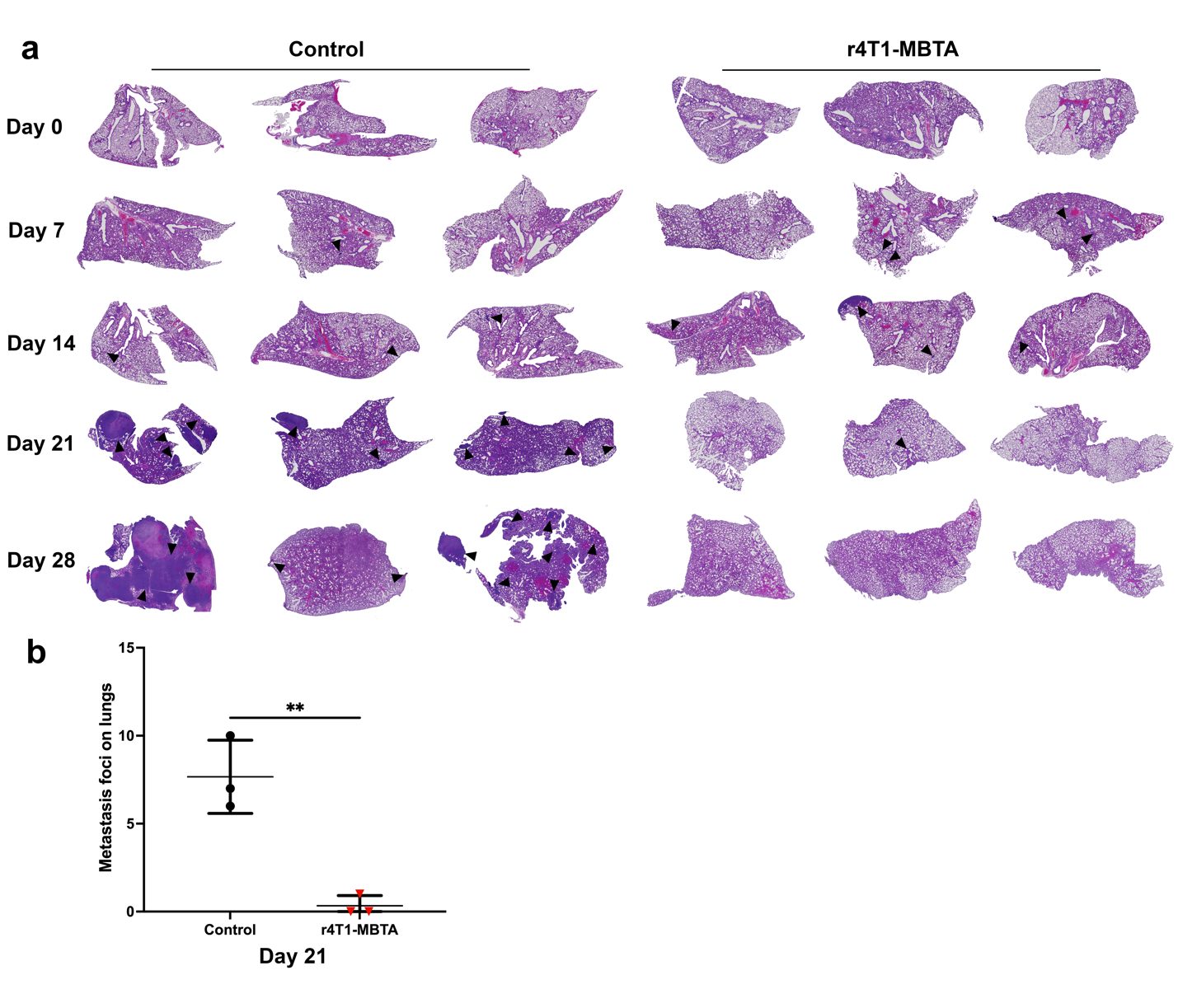


**Supplementary Figure 3. H&E staining of lung sections collected for 4T1 dynamic tumor metastasis from tumor removal animal model** (Corresponding to figure 3) **a,** Representative H&E staining of the lung tissue collected each week after surgery from 4T1 tumor removal animal model. The black arrows indicate tumor metastasis foci (n=3).

 **b,** Quantification number of metastatic nodes in the lungs of mice on day 21 of the study (n=3/group). Unpaired T-test was used to assess statistical significance. All data are represented as mean ± SD. *P*-values were shown for control versus the rWTC-MBTA vaccine. **P*<0.05, ***P*<0.01,****P*<0.001,**** *P*<0.0001.



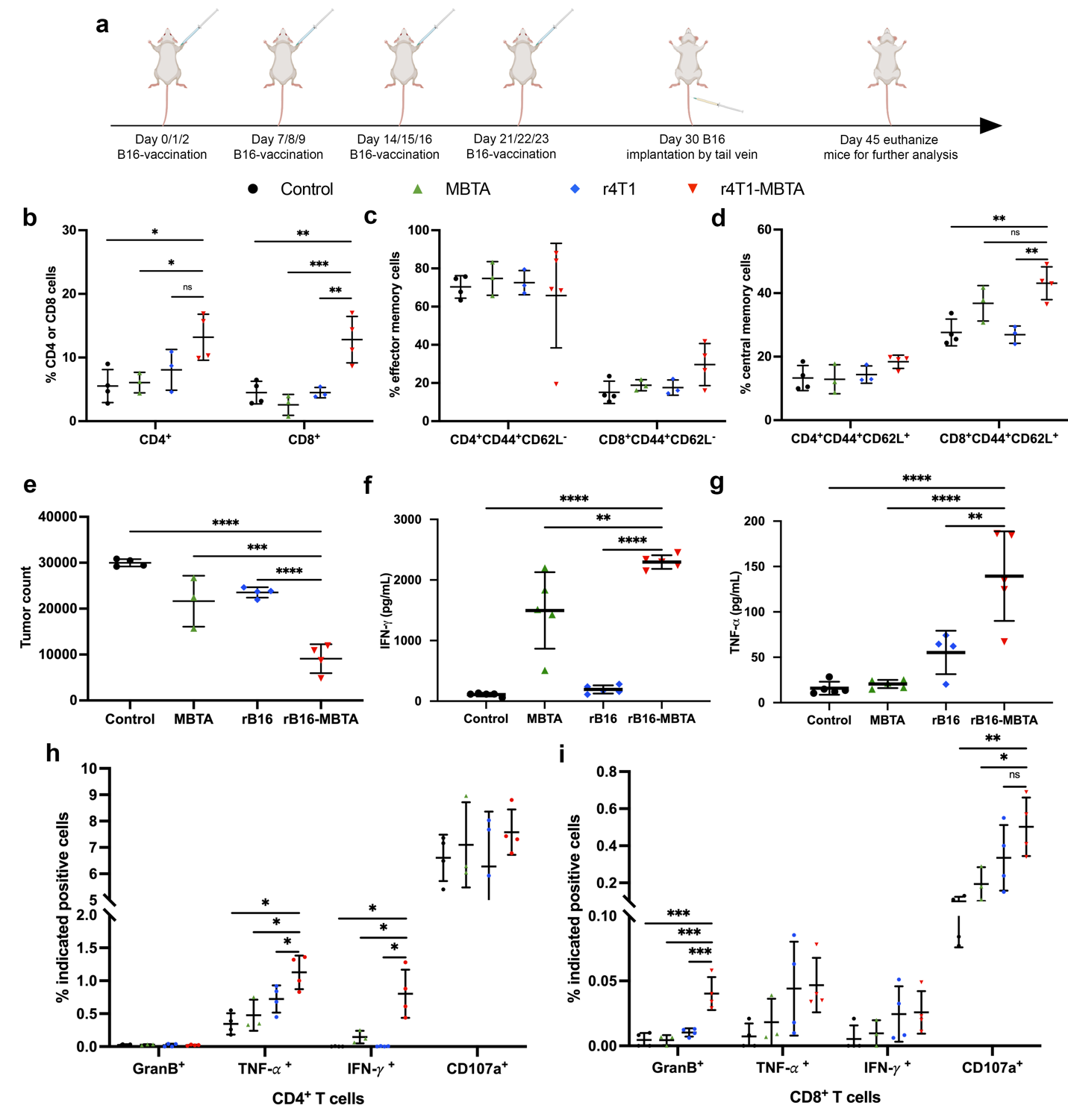


**Supplementary figure 4. Enhanced adaptive and memory immunity in melanoma model after vaccine treatment**. Spleens were collected and isolated at the endpoint of the B16 subcutaneous metastasis animal model (**a**). Immune CD4^+^ and CD8^+^ T cells were examined by flow cytometry in splenocytes from different treatment groups. **b,** Percentage of CD4 and CD8 T cells in splenocytes. **c,** Percentage of effector memory (CD44^+^CD62L^-^) T cells in splenocytes. **d,** Percentage of central memory (CD44^+^CD62L^+^) T cells in splenocytes of different treatment groups. **e-i,** All the analyses are based on the co-culture B16 tumor cells and indicated splenocytes from different treated animals. **e,** 4T1 tumor cell number count after co-culture. **f & g,** Concentrations of cytokines IFN-𝛾 (**f**) and TNF-𝛼 (**g**) from co-culture supernatants as measured by ELISA. **h&i,** the percentage of Gran B^+^, IFN-𝛾^+^, TNF-𝛼^+^, or CD107^+^ CD4^+^ or CD8^+^ T cells of total CD45+ cells in each group was determined by flow cytometry. The analysis from **e** to **i** was conducted after co-culturing splenocytes (from different treated mice) with 4T1 tumor cells. Ordinary one-way ANOVA with multiple comparisons was used to assess statistical significance. All data are represented as mean ± SD. *P*-values were shown for each treated group (control, MBTA only, or rWTC only group) versus the rWTC-MBTA vaccine (n≥3/group). **P*<0.05, ***P*<0.01, ****P*<0.001, **** *P*<0.0001.


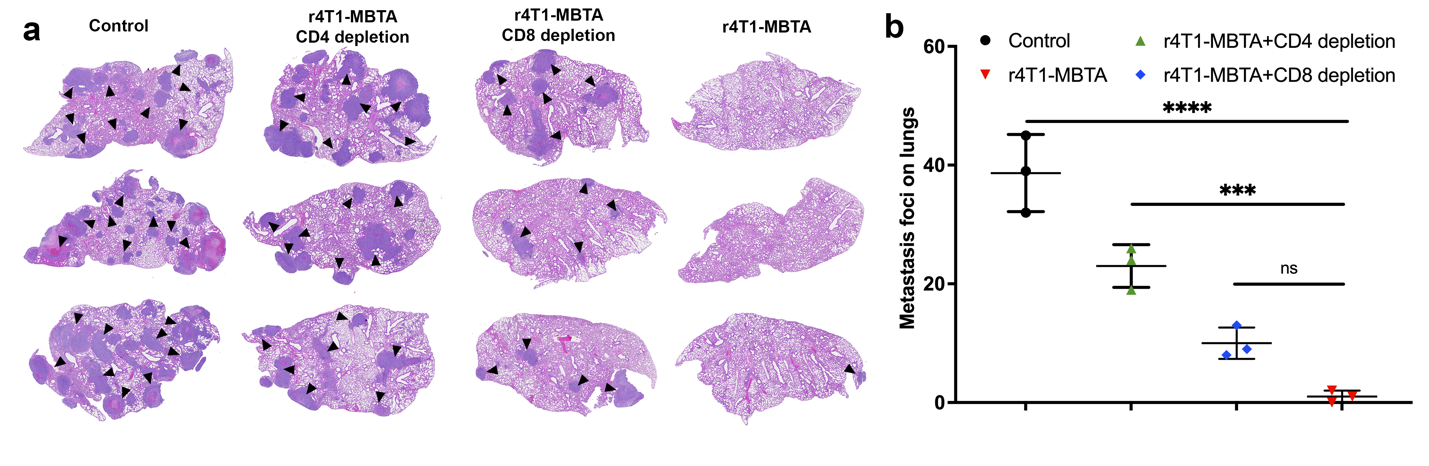


**Supplementary Figure 5. H&E staining of lung sections collected for tumor metastasis from T cell depletion animal model** (corresponding to figure 7). **a**, Representative H&E staining of the lung tissue collected from the T cell depletion animal model. The black arrows indicate tumor metastasis focus. **b**, Quantification of lung metastasis nodes from T cell depletion mice. Ordinary one-way ANOVA with multiple comparisons was used to assess statistical significance. All data are represented as mean ± SD. *P*-values were shown for each treated group (control, MBTA only, or rWTC only group) versus the rWTC-MBTA vaccine (n=3/group). **P*<0.05, ***P*<0.01, ****P*<0.001, **** *P*<0.0001.


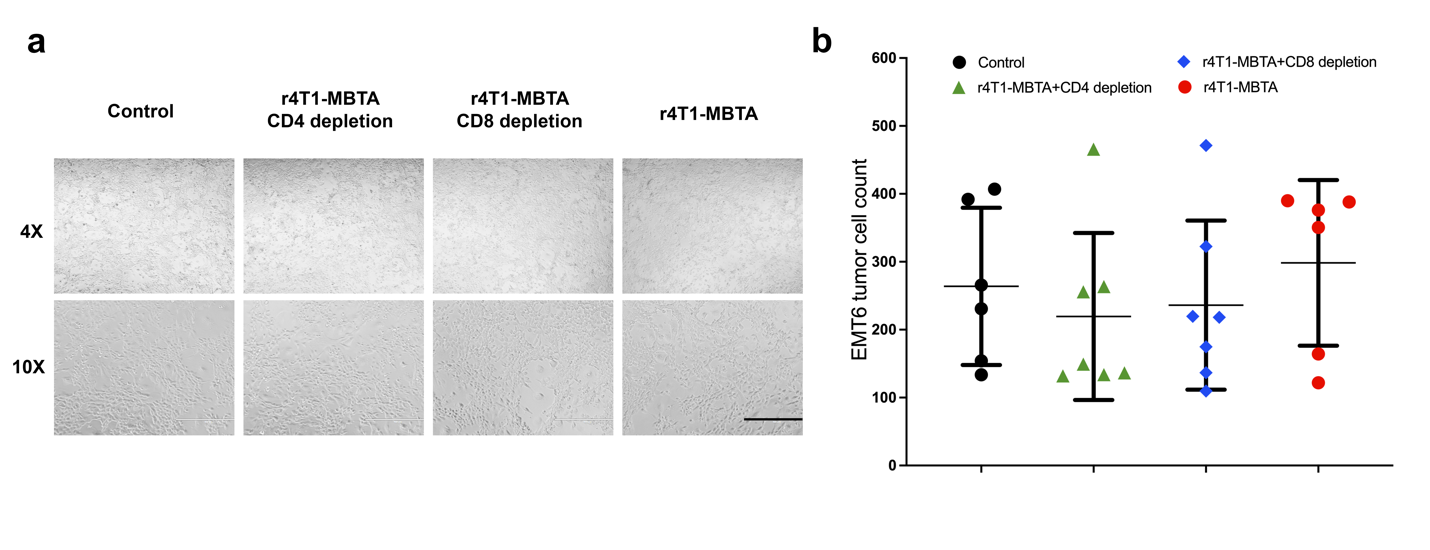


**Supplementary Figure 6. T cell depletion demonstrates cytotoxicity of T cells from r4T1-MBTA vaccinated mice against autologous but not allogeneic tumor cells. a,** Representative photographs 48 hours after co-culture of T-cell and EMT6 tumor cell (scale bar= 400 nm). **b**, Tumor cell counting by flow cytometry 48 hours after co-culture of T-cell and 4T1 tumor cells. Ordinary one-way ANOVA with multiple comparisons was used to assess statistical significance. All data are represented as mean ± SD. *P*-values are for each treated group (control, r4T1-MBTA+CD4 depletion, or r4T1-MBTA+CD8 depletion gp) versus the rWTC-MBTA vaccine (n≥5/group).
