# Supplementary material for: rWTC-MBTA: autologous vaccine prevents metastases via antitumor immune responses
Source: J Exp Clin Cancer Res. 2023 Jul 12;42:163. doi: 10.1186/s13046-023-02744-8 (PMC10337177; doi:10.1186/s13046-023-02744-8)
Supplement: Supplementary file 3 — Supplementary Material 3 [file 13046_2023_2744_MOESM3_ESM.docx]

We appreciate all the valuable and insightful comments from the reviewers of our work. We have revised our manuscript, according to the reviewers’ comments, questions, and suggestions. We believe that the manuscript has been further improved. Here are the major revisions:

Reviewer reports:

Reviewer #1: Paper by Juan Ye et al. showed results indicating that  irradiated tumor cells (rWTC) pulsed with mannan-BAM, TLR agonists, and anti-CD40 antibody (MBTA), are able to prevent tumor metastasis in multiple animal models.
Despite of authors have conducted a number of convincing experiments demonstrating in animal models the activity in preventing metastasis, I think that this paper suffer of a major drawback that must be  carefully addressed by authors.
From introduction and throughout all the paper Authors stated that  "we propose an innovative approach utilizing immunotherapy. Our strategy involves utilizing an   autologous whole tumor cell vaccine, rWTC-MBTA, which is composed of irradiated entire tumor  cells (rWTC) pulsed with mannan-BAM, TLR agonists, and anti-CD40 antibody (MBTA)"
This strategy is not particularly innovative  and we already know its utilization in solid tumours ( as an example see review Int. J. Mol. Sci. 2021, 22, 3455. <https://gcc02.safelinks.protection.outlook.com/?url=https%3A%2F%2Fdoi.org%2F10.3390%2Fijms22073455&data=05%7C01%7Czhengping.zhuang%40nih.gov%7C0b66584366f24ab996d508db51694e7d%7C14b77578977342d58507251ca2dc2b06%7C0%7C0%7C638193284957199302%7CUnknown%7CTWFpbGZsb3d8eyJWIjoiMC4wLjAwMDAiLCJQIjoiV2luMzIiLCJBTiI6Ik1haWwiLCJXVCI6Mn0%3D%7C3000%7C%7C%7C&sdata=fIhTvjzN4RGiKwKolEGkH4e4Kd6johIxZi0Q9lFgFi8%3D&reserved=0> or Adv. Ther. 2020, 3, 2000044, the last one cited by authors), therefore I think that authors have to  better explain the novelty of their paper  focusing introduction on metastasis treatment. In this regard even in discussion authors should dicuss their results enlightening difference or similarity in the mechanisms against metastasis respect what we already know about mechanism of action of irradiated entire tumor  cells pulsed with mannan-BAM, TLR agonists, and anti-CD40 antibody.
In conclusion, paper is of some interest  but requires profound changes in introduction and discussion.

Thank you, reviewer#1, for your insightful comments and suggestions. We agree that we did not clarify the difference between this work and our previous work (*Medina* et al. 2020). We have made significant changes to the introduction and discussion to make it clear that in our previous work we only sought to determine the therapeutic efficacy of rWTC-MBTA in a mouse colon cancer model, however at the time we did not determine its efficacy as a preventative metastatic vaccine, nor did we elaborate on the mechanism to by which rWTC-MBTA confers protection. In this study we demonstrate that rWTC-MBTA can prevent tumor metastasis in several animal models, which has previously not been demonstrated. Furthermore, we build upon on previous study and further elucidate on the mechanisms that protect against tumor metastasis with our T-cell and APC analysis. As per your suggestion we have made major changes to the discussion and introduction to make it clear that our vaccine strategy is not novel per se but rather builds upon our previous study by showing its efficacy as a preventative metastatic vaccine, builds upon the mechanistic studies, and better understand its safety profile.

Reviewer #2: The authors tested whether their autologous vaccine (rWTC-MBTA) built by combining irradiated tumor cells, a bacterial PAMP (Mannan-BAM), TLR agonist and anti-CD40 mAbs  could prevent development of experimental metastases in different immunocompetent pre-clinical models. In  4T1 breast cancer and B16 melanoma models vaccination reduced frequency of metastatic lungs  after tumor challenge. The protecton against development of experimental metastases was retained even by a tumor challenge at day+ 90 after the end of vaccination.   In a different, post-surgical vaccination experiment expansion of CD4 and CD8 T cells was observed in the lungs of vaccinated animals and survival of vaccinated mice was significantly prolonged. Moreover challenge with tumor  in surviving mice at T=1 year led to markedly reduced tumor growth. They then show that vaccination inhibited only growth, as well as lung mets, after a subsequent challenge with autologous but not with an
allogeneic tumor.  Vaccination led at day 26 to a significant increase in DC, monocytes and B cells in the lymph nodes of treated mice. Serum of vaccinated mice also showed a significant increase in IFN-G and IL-6 levels. Spleens of vaccinated mice contained higher frequencies of CD4 and CD8 T cells  with a strong increase in effector memory and central memory subsets.  Co-culture experiments with immune cells and tumor cells led to a reduction in the tumor cell counts and to release in the supernatants of IFNg. By flow cytometry they show increase of T cells expressiong TNFa, IFNG and CD107a among splenocytes of vaccinated mice compared to controls. Experiments inducing depletion of CD4 or CD8 T cells in vaccinated mice did not prevent development of experimental lung mets after tail vein injection of tumor cells. The effect of depletion on lung mets development was more pronounced for CD4 than for the CD8 subsets. Finally, assessment of markers of toxicity associated with
the vaccination procedure did not document significant changes in several established blood parameters  linked to hepatotoxicity or to nephrotoxicity. Histological analysis did not reveal major pathological changes in main organs.


Specific comments
Fig 2. Panels d and g: it would be better to have plots showing the actual number of lung mets in each mouse, instead of floating bars. The same is true for figure 4 D and H. Also the legend should mention how many mice/group were treated. In addition results of the multiple comparison tests should be shown for each group allowing to visualize the significance of differences among any of the four treatment groups. This is relevant since even vaccination with unmodified tumor cells seems effective. The point here is to what extent the tumor plus mannan-BAM, TLR agonists and anti-CD40 is more effective compared to tumor alone.
Fig. 3b. The author should plot the number of spontaneous mets in control vs vaccinated animals at least at day +21.

We are grateful to review #2 for their insightful comments our manuscript. We have been able to incorporate changes to reflect most of the suggestions provided by the reviewers. Here is a point-by-point response to the reviewers' comments and concerns:

1. We have changed most of the graphs to show individual mice versus floating bars, not just the ones suggested by the reviewer.
2. We have added in the figure legend the number of mice per group.
3. For Fig. 4 panels D and H, the multiple comparisons test between groups has been added as suggested by the reviewer. We agree that the comparison between rWTC-MBTA, MBTA alone, and rWTC is important.
4. Regarding Fig 3b, we have conducted the quantification of spontaneous metastases in the control group versus the vaccinated group on day 21, as shown in Supplementary Fig 3b. Unfortunately, on day 24, the tumor lesions in the control group where numerous taking up most if not all the lung, making it impossible to quantify the metastases. Therefore, we focused solely on quantifying the spontaneous metastases on day 21.

Editor's comments: The paper uses various animal models that undoubtedly cause suffering, has the ethics of the experimental procedures been carefully evaluated even beyond the simple assumption that the Ethics Committee of the Institute proposing the study approved these procedures?

We understand the editor's concerns about the ethical considerations surrounding our use of animal models in the study. We want to assure the editor that we took the ethical aspects very seriously. Before starting out study, our mouse protocol underwent a thorough evaluation by the Ethics Committee of National Cancer Institutes. This committee consists of experts who specialize in animal welfare and ensure that all proposed procedures comply with established ethical guidelines. We made every effort to minimize any potential suffering experienced by the animals involved. We followed internationally recognized guidelines for the care and use of laboratory animals, which included providing suitable living conditions, appropriate veterinary care, and using humane endpoints to ensure the animals' well-being. Our research team received extensive training in handling animals and conducting experiments to ensure that we maintained the highest standards of animal welfare. We also tried to use the minimum number of animals necessary to obtain reliable results, while considering alternatives to animal use whenever possible. In the whole project, we are fully committed to conducting research in an ethical and responsible manner, following established guidelines and regulations for the use of animal models.
